# Uncoupling FoxO3A mitochondrial and nuclear functions in cancer cells undergoing metabolic stress and chemotherapy

**DOI:** 10.1038/s41419-018-0336-0

**Published:** 2018-02-14

**Authors:** Valentina Celestini, Tugsan Tezil, Luciana Russo, Candida Fasano, Paola Sanese, Giovanna Forte, Alessia Peserico, Martina Lepore Signorile, Giovanna Longo, Domenico De Rasmo, Anna Signorile, Raffaella Maria Gadaleta, Natasha Scialpi, Mineko Terao, Enrico Garattini, Tiziana Cocco, Gaetano Villani, Antonio Moschetta, Valentina Grossi, Cristiano Simone

**Affiliations:** 1 Division of Medical Genetics, Department of Biomedical Sciences and Human Oncology (DIMO), University of Bari Aldo Moro, Bari, 70124 Italy; 2 Department of Biochemistry and Molecular Pharmacology/Laboratory of Molecular Biology, IRCCS - Istituto di Ricerche Farmacologiche ‘Mario Negri’, Milano, 20156 Italy; 3 Medical Genetics, National Institute for Gastroenterology, IRCCS ‘S. de Bellis’, Castellana Grotte (Ba), 70013 Italy; 4 Department of Molecular Medicine, Sapienza University of Rome, 00161 Rome, Italy; 5 Institute of Biomembranes and Bioenergetics, National Research Council (CNR), Bari, 70126 Italy; 6 Department of Basic Medical Sciences, Neurosciences and Sense Organs, University of Bari Aldo Moro, Bari, 70124 Italy; 7 Division of Digestive Diseases, Department of Surgery and Cancer, Imperial College London, Queen Elizabeth the Queen Mother Wing (QEQM), London, W2 1NY UK; 8 Medicina Interna Universitaria Frugoni’, Department of Interdisciplinary Medicine, University of Bari Aldo Moro, Bari, 70124 Italy

## Abstract

While aberrant cancer cell growth is frequently associated with altered biochemical metabolism, normal mitochondrial functions are usually preserved and necessary for full malignant transformation. The transcription factor FoxO3A is a key determinant of cancer cell homeostasis, playing a dual role in survival/death response to metabolic stress and cancer therapeutics. We recently described a novel mitochondrial arm of the AMPK-FoxO3A axis in normal cells upon nutrient shortage. Here, we show that in metabolically stressed cancer cells, FoxO3A is recruited to the mitochondria through activation of MEK/ERK and AMPK, which phosphorylate serine 12 and 30, respectively, on FoxO3A N-terminal domain. Subsequently, FoxO3A is imported and cleaved to reach mitochondrial DNA, where it activates expression of the mitochondrial genome to support mitochondrial metabolism. Using FoxO3A^−/−^ cancer cells generated with the CRISPR/Cas9 genome editing system and reconstituted with FoxO3A mutants being impaired in their nuclear or mitochondrial subcellular localization, we show that mitochondrial FoxO3A promotes survival in response to metabolic stress. In cancer cells treated with chemotherapeutic agents, accumulation of FoxO3A into the mitochondria promoted survival in a MEK/ERK-dependent manner, while mitochondrial FoxO3A was required for apoptosis induction by metformin. Elucidation of FoxO3A mitochondrial vs. nuclear functions in cancer cell homeostasis might help devise novel therapeutic strategies to selectively disable FoxO3A prosurvival activity.

## Introduction

Carcinogenesis is a multistep process by which normal cells evolve to a neoplastic state by acquiring a succession of cancer hallmarks^1^. Tumor cell homeostasis is sustained by the balance between these newly acquired oncogenic features and pre-existing cellular functions. Paradigmatic in this regard is the reprogramming of energy metabolism, where normal cellular processes providing increased energy production, macromolecular biosynthesis, and redox balance maintenance^2–4^ are ensured by the preservation of key mitochondrial functions^5–7^.

Consistent with this view, proteins that have been classically considered as tumor suppressors are sometimes required to be functional for full malignant transformation. This is the case for FoxO3A, which can be both friend and foe to cancer cells depending on the cellular context^8–10^.

FoxO3A belongs to the FoxO (Forkhead-box O) family of transcription factors, together with FoxO1, FoxO4 and FoxO6, which are evolutionarily conserved from nematodes to mammals^11^. In mammals, FoxO3A functions are mediated by the activation of a coordinated transcriptional program involving genes that regulate cell cycle control, cell death, cell metabolism, redox balance, DNA repair and autophagy^8^. As all these genes share the conserved consensus core recognition motif FHRE (5′TTGTTTAC3′) within their DNA regulatory regions, expression specificity is ensured by additional regulation mechanisms such as phosphorylation-dependent subcellular localization, whereby some kinases trigger FoxO3A nuclear exclusion and subsequent cytoplasmic degradation (AKT and IKKß) and others enable its nuclear localization and transcriptional activation (p38 and AMPK)^12–14^. These enzymes define the so-called “molecular FOXO code”, which is critical for the fine-tuned regulation of FoxO factors’ different functions. FoxO3A has emerged as a major sensor for metabolic stress and chemotherapeutic drug response in cancer cells, playing a dual role at the crossroad between survival and death. In metabolically stressed cancer cells, activation of the FoxO3A-dependent transcriptional program first leads to autophagy and cell cycle arrest as an attempt to retain energy and increase ATP levels to survive, but then triggers cell death under persistent stress conditions^15–17^. Consistently, in cancer cells undergoing therapy-induced genotoxic stress, FoxO3A is involved in detoxification and DNA repair thereby promoting survival, while its pro-apoptotic function likely reflects an irreparable level of damage^18,19^.

Recently, we reported that glucose restriction causes the AMPK-dependent accumulation of FoxO3A into the mitochondria of normal fibroblasts and muscle cells in culture, followed by the formation of a transcriptional complex containing FoxO3A, SIRT3 and the mitochondrial RNA polymerase (mtRNAPOL) at mitochondrial DNA regulatory regions, thereby promoting expression of the mitochondrial genome and a subsequent increase in oxygen consumption. These results were confirmed *in vivo* in tissues of fasting mice^20^, thus revealing a mitochondrial arm of the AMPK-FoxO3A axis operating as a recovery mechanism to sustain cellular metabolism upon nutrient shortage and metabolic stress.

Here, we characterize this novel FoxO3A function in cancer cells and provide compelling molecular evidence that in metabolically stressed cancer cells and tumors FoxO3A is recruited to the mitochondrial surface in a MEK/ERK- and AMPK-dependent manner, while only the MEK/ERK signal is required in cancer cells treated with chemotherapeutic agents. After cleavage of its N-terminal domain, FoxO3A is imported into the mitochondrial matrix where it activates a transcriptional program leading to cancer cell survival. On the other hand, mitochondrial FoxO3A (mtFoxO3A) is required for apoptosis induction in cancer cells treated with metformin.

## Results

### FoxO3A is cleaved at its N-terminus upon translocation into the mitochondria of normal and cancer cells

**Fig. 1:**
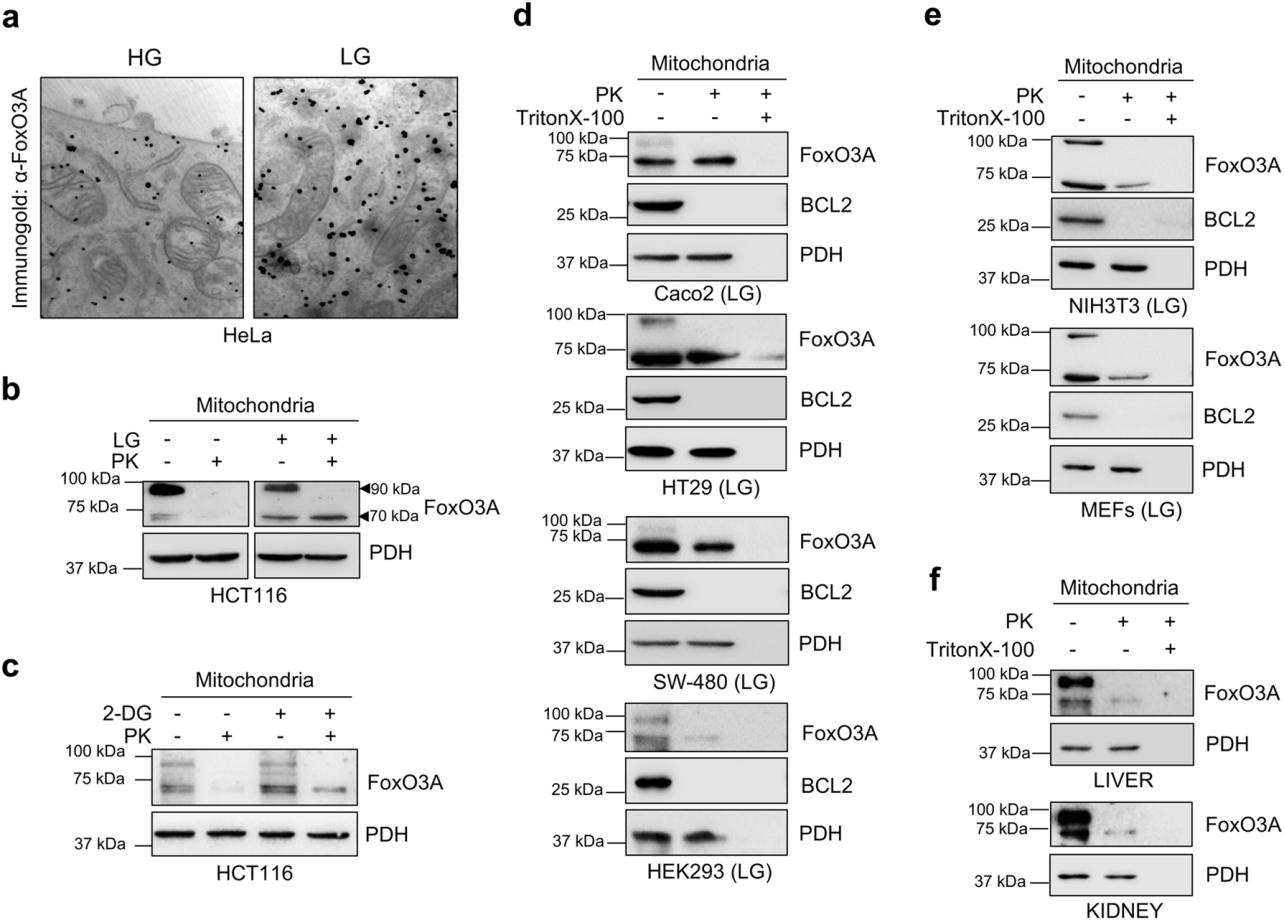
FoxO3A accumulates into the mitochondria in metabolically stressed cell lines and normal tissues. **a** Immunogold labeling of HeLa cells cultured in high glucose (HG) or switched to low glucose (LG, 0.75 mM glucose) for 24 h. Black dots represent gold particles recognizing FoxO3A immunocomplexes. **b**,** c** Immunoblot analysis of mitochondria isolated from HCT116 cells upon **b** LG (24 h) and **c** 2-deoxy-glucose (2-DG) treatment (1 mM, 6 h). Mitochondrial fractions were treated with proteinase K (PK) to degrade outer mitochondrial membrane proteins. PDH: loading control. **d**, **e** Immunoblot analysis of mitochondrial fractions isolated from Caco2, HT29, SW-480 and HEK293 (**d**) and from NIH3T3 and MEF murine fibroblasts (**e**) cultured in LG (24 h). Mitochondrial fractions were treated with PK alone or with PK and Triton X-100 to permeabilize mitochondria and degrade all mitochondrial proteins. BCL2: outer membrane control; PDH: mitochondrial matrix control. **f** Immunoblot analysis of mitochondria-enriched fractions isolated from murine kidney and liver and subjected to PK or combined PK and Triton X-100 treatment. PDH: mitochondrial matrix control. The presented results are representative of at least three independent experiments

In line with our previous findings in human and rodent normal cells and tissues^20^, we detected FoxO3A into the mitochondria of metabolically stressed HeLa cancer cells (Fig. 1a).

To ascertain whether it could undergo post-translational processing similar to other nuclear-encoded mitochondrial proteins^21^, we purified mitochondria from several normal and cancer cell lines of different origin cultured under high (HG) or low (LG) glucose conditions and performed a proteinase K (PK) protection assay to detect proteins localized at the outer membrane or inside the mitochondria. In LG, anti-FoxO3A antibodies revealed the presence of two bands in the whole mitochondria fraction (at around 90 and 70 kDa), while after PK treatment only the 70 kDa band was detected into the organelles (Fig. 1b, d, e and Supplementary Figure S1b). These results indicate that anti-FoxO3A antibodies recognized the full-length protein (90 kDa band) bound outside of the mitochondria, while inside the organelle they detected a shorter 70 kDa form. These findings were confirmed by treating cells with 2-deoxy-glucose (2-DG) or iodoacetic acid (IA), two diverse glycolysis inhibitors that induce metabolic stress (Fig. 1c and Supplementary Figure S1a). Of note, a similar migration pattern was observed *in vivo* when analyzing mitochondria isolated from different mouse tissues (Fig. 1f and Supplementary Figure S1c).

**Fig. 2:**
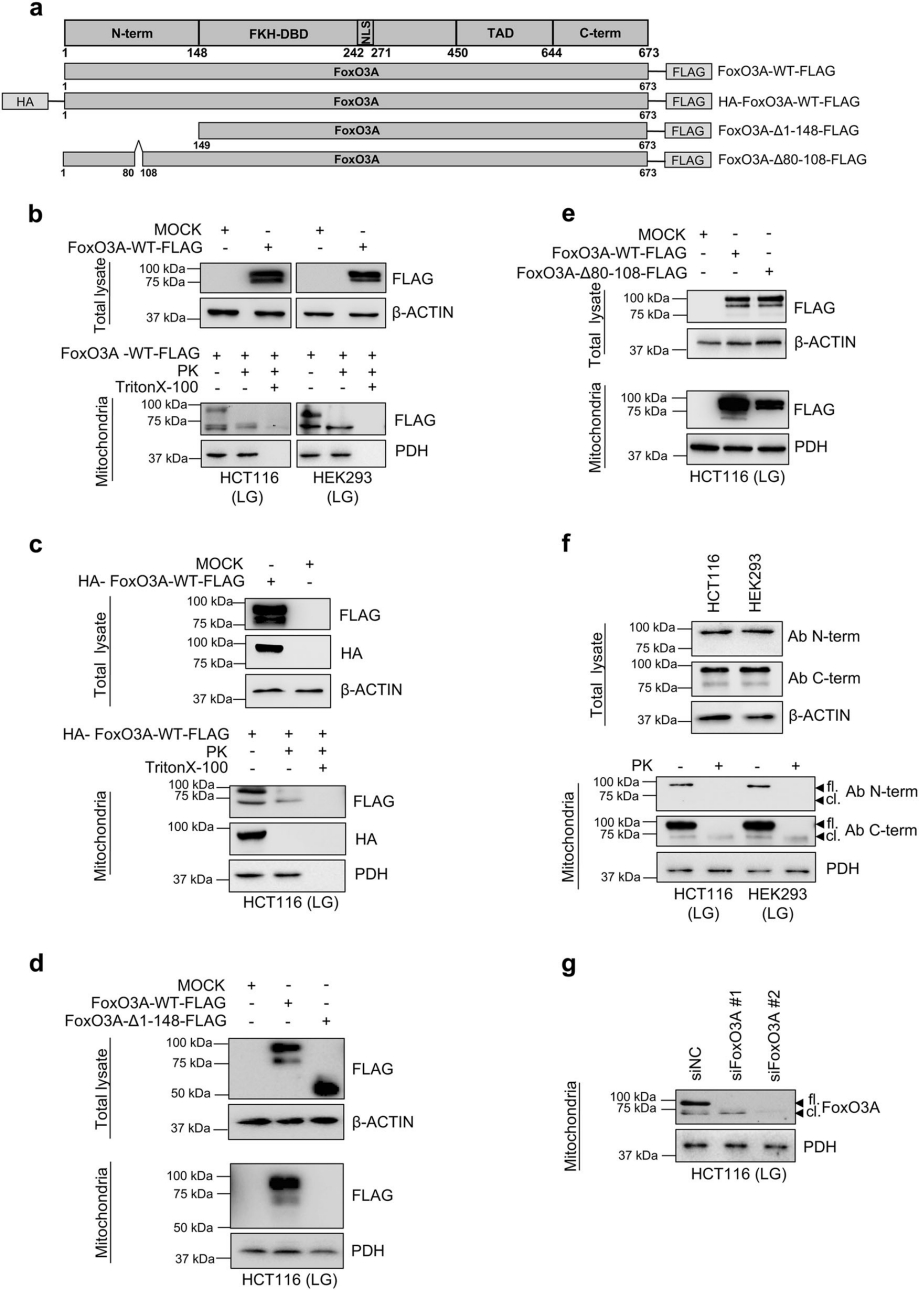
FoxO3A is cleaved at its N-terminus upon translocation into the mitochondria of normal and cancer cells. **a** Scheme of plasmids used. **b–e** Cells were transfected with the indicated FoxO3A plasmids for 48 h; upon LG (0.75 mM glucose, 24 h), purified mitochondria were treated with PK alone or with PK and Triton X-100. Total and mitochondrial proteins were analyzed by immunoblot. **b** HCT116 and HEK293 cells transfected with FoxO3A-WT-FLAG. **c** HCT116 cells transfected with HA-FoxO3A-WT-FLAG. **d** HCT116 cells transfected with FoxO3A-WT-FLAG or FoxO3A-Δ1–148-FLAG. **e** HCT116 cells transfected with FoxO3A-WT-FLAG or FoxO3A-Δ80-108-FLAG. β-actin and PDH were used as total lysate and mitochondria controls, respectively. **f** Immunoblots performed with two different anti-FoxO3A antibodies in mitochondria isolated from HCT116 and HEK293 cells cultured in LG (24 h). Mitochondrial fractions were subjected to PK treatment. β-actin and PDH were used as total lysate and mitochondrial fraction controls, respectively. **g** HCT116 cells were transfected with control (siNC) or FoxO3A-specific siRNAs for 48 h and FoxO3A mitochondrial levels were evaluated by immunoblot upon LG (24 h). PDH: loading control. fl. full-length FoxO3A, cl. cleaved FoxO3A, N-term. N-terminal domain, FKH-DBD forkhead DNA-binding domain, NLS nuclear localization signal, TAD transactivation domain, C-term. C-terminal domain. The presented results are representative of at least three independent experiments

To our knowledge, this is the first report describing a shorter form of FoxO3A and suggests that FoxO3A may be processed similar to canonical mitochondrial proteins. To rule out non-specific antibody binding, we transfected a vector-expressing FoxO3A FLAG-tagged at the C-terminal domain (Fig. 2a) in glucose-restricted HCT116 and HEK293 cells. Anti-FLAG antibodies revealed two bands (90 and 70 kDa) corresponding to exogenous FoxO3A in the purified mitochondrial fraction, while only the 70 kDa band was detected after addition of PK (Fig. 2b). Consistently, glucose-restricted HCT116 cells transfected with a newly generated vector-expressing FoxO3A tagged both at the N-terminus (with HA) and at the C-terminus (with FLAG) showed the presence of both signals (HA and FLAG) at 90 kDa in purified mitochondria, while only a 70 kDa band was detected by anti-FLAG antibodies after PK treatment (Fig. 2c). Finally, we deleted the entire N-terminus (aa 1–148) from FoxO3A-FLAG and assessed its ability to localize at the mitochondria. Our data indicate that amino acids 1–148 are required for FoxO3A localization at, and translocation into the mitochondria (Fig. 2d).

To further characterize FoxO3A N-terminus, we searched for mitochondrial processing peptidase (MPP) and mitochondrial intermediate peptidase (MIP) consensus motifs and found partially overlapping R-2, R-3, and R-10 motifs^22^ in sequence ARVLAPGGQD of FoxO3A region 98–108 (Supplementary Figure S2a). Thus, we generated a FLAG-tagged vector encoding for FoxO3A-Δ80-108 and found that it was able to localize at -but impaired in translocating into- the mitochondria, as shown by the absence of the 70 kDa band (Fig. 2e).

We then purified mitochondria from metabolically stressed HCT116 and HEK293 cells and performed an immunoblot analysis of endogenous FoxO3A with two antibodies, one directed against the N-terminus, which only detected a single band of around 90 kDa in the whole mitochondria fraction, and the other targeting the C-terminal domain, which recognized both the 90 kDa and the 70 kDa bands in whole mitochondria and only the 70 kDa band inside the organelles (Fig. 2f). Moreover, genetic silencing using two FoxO3A-specific siRNAs confirmed that the 70 kDa band detected inside the mitochondria is a shorter form of endogenous FoxO3A (Fig. 2g). Further PK protection assays confirmed that endogenous FoxO3A is translocated into the mitochondria and reaches the matrix in its cleaved 70 kDa form (FoxO3A-cl.) (Supplementary Figure S2b, S2c). These results were corroborated by immunoblot analysis of mitoplasts from swollen mitochondria treated with PK (Supplementary Figure S2b, S2d).

These data indicate that FoxO3A N-terminus (aa 1–148) is required for proper recruitment to the mitochondria, with residues 98–108 being necessary for FoxO3A cleavage and import into the mitochondrial matrix. Interestingly, this region is specific to FoxO3A (it is not conserved in other human FoxO members) (Supplementary Figure S3), and is evolutionarily conserved across species (Supplementary Figure S4b).

### Phosphorylation at S12 and S30 of FoxO3A N-terminus is required for mitochondrial accumulation in metabolically stressed cancer cells

FoxO3A N-terminus (aa 1–148) has been poorly characterized, except for threonine 32, which represents a well-known target of AKT^13^. Thus, we searched for novel residues that could be targeted by signaling pathways transducing extracellular stimuli. Based on our in silico analysis with NETPHOS 2.0 and DISPHOS 1.3 servers, only six serines (12, 26, 30, 43, 48 and 55) reached a significant threshold score (>0.6) with both tools (Supplementary Table S1). Furthermore, all these residues have been described as being phosphorylated *in vivo* in different types of human cancers^23–29^.

**Fig. 3:**
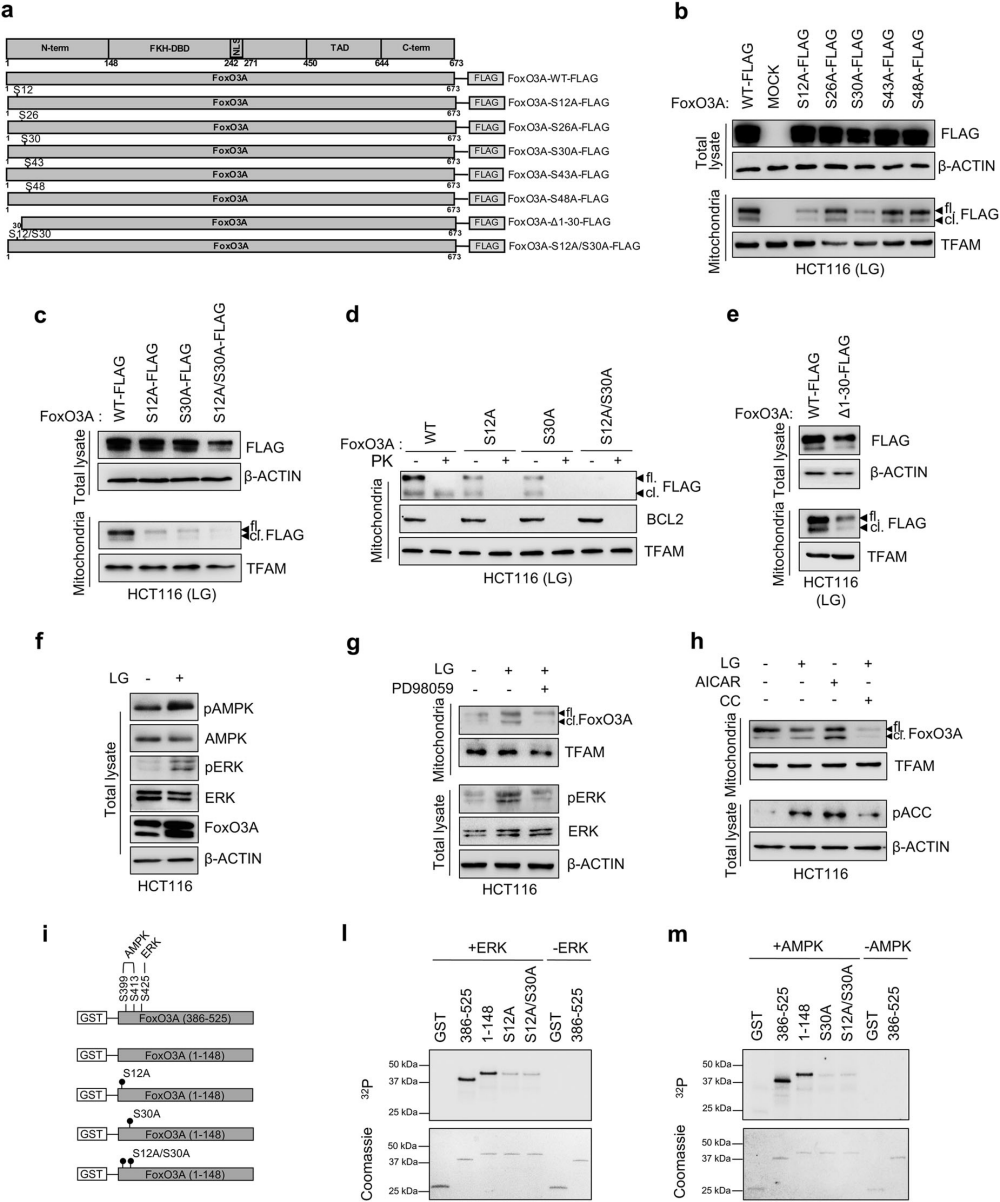
Phosphorylation at S12 and S30 of FoxO3A N-terminus is required for mitochondrial accumulation in metabolically stressed cancer cells. **a** Scheme of FoxO3A-WT-FLAG and mutated FoxO3A-FLAG plasmids obtained by site-directed mutagenesis. **b**,** c** HCT116 cells were transfected with the indicated FoxO3A-WT-FLAG and mutant plasmids for 48 h. Upon LG (0.75 mM glucose, 24 h), total and mitochondrial proteins were analyzed by immunoblot. **d** HCT116 cells were transfected with the indicated FoxO3A-WT-FLAG and mutant plasmids for 48 h. Upon LG (24 h), purified mitochondrial fractions were treated with PK and analyzed by immunoblot. BCL2 outer membrane control, TFAM mitochondrial matrix control. **e** HCT116 cells were transfected with FoxO3A-WT-FLAG or FoxO3A-Δ1-30-FLAG for 48 h, cultured in LG (24 h). **f** Total proteins were isolated from HCT116 cells grown in standard and LG (24 h) conditions and analyzed by immunoblot. **g** Pharmacological inhibition of MEK using PD98059 in HCT116 cells cultured in LG (24 h). Total and mitochondrial proteins were analyzed by immunoblot. **h** Pharmacological inhibition or activation of AMPK using compound C (CC, 5 μM) or AICAR (5 mM), respectively, in HCT116 cells cultured in LG (24 h). Total and mitochondrial proteins were analyzed by immunoblot. **b**,** c**,** e**–**h** β-actin and TFAM were used as total lysate and mitochondrial fraction controls, respectively. **i** Scheme of GST-FoxO3A recombinant proteins. **l**,** m** *In vitro* kinase assays performed with the indicated GST-FoxO3A(1–148) and mutant recombinant proteins as substrates, in the presence of recombinant ERK (**l**) or recombinant AMPK (**m**). In both assays, GST-FoxO3A(386–525) was used as a positive control. GST-empty protein was used as a negative control. Coomassie gel staining (lower panels) was used as a loading control. fl. full-length FoxO3A, cl. cleaved FoxO3A, N-term. N-terminal domain, FKH-DBD forkhead DNA-binding domain, NLS nuclear localization signal, TAD transactivation domain, C-term. C-terminal domain. The presented results are representative of at least three independent experiments

To test whether these serines were involved in FoxO3A accumulation into the mitochondria, we mutagenized them to non-phosphorylatable alanine residues (Fig. 3a and Supplementary Figure S5a) and expressed the indicated FoxO3A FLAG-tagged proteins in glucose-restricted cancer cells. Our data show that alanine substitution at position 12 or 30 impaired the ability of full-length FoxO3A (FoxO3A-fl.) to be recruited at the outer membrane and to localize inside the mitochondria in its cleaved form (FoxO3A-cl.) (Fig. 3b). Furthermore, concomitant substitution of both serines (S12A/S30A) completely abrogated FoxO3A mitochondrial localization (Fig. 3c, d), suggesting that S12 and S30 are both required for FoxO3A translocation into the mitochondria in metabolically stressed cancer cells. These data were confirmed with a vector encoding for a FoxO3A mutant lacking the initial 30 residues of the N-terminal domain [FoxO3A-Δ1-30-FLAG], which was found to be severely impaired in mitochondrial localization (Fig. 3e).

Since AMPK is involved in FoxO3A mitochondrial accumulation in normal cells under glucose deprivation^20^, we performed a comparative analysis between well-known AMPK consensus phosphorylation motifs and the amino acid sequence surrounding FoxO3A serines located at the N-terminal domain. Our findings suggest that S30 could represent a novel AMPK phosphorylation site in FoxO3A (Supplementary Table S2). Likewise, Kinasephos 2.0 analysis predicted that S12 could be part of an ERK consensus phosphorylation motif (Ser-Pro). Thus, we characterized glucose-restricted HCT116 cells and observed that both AMPK and ERK kinases were phospho-activated in these culture conditions (Fig. 3f) and could be localized at the mitochondria together with FoxO3A (Supplementary Figure S5b). Of note, pharmacological inhibition of the MEK/ERK or the AMPK pathway by PD98059 or compound C (CC), respectively, significantly prevented FoxO3A localization at (FoxO3A-fl.) and accumulation into (FoxO3A-cl.) the mitochondria (Fig. 3g, h). Furthermore, activation of AMPK by AICAR significantly induced FoxO3A mitochondrial processing and import, as shown by its cleaved form levels, even in HG culture conditions (Fig. 3h).

To confirm the hypothesis that S12 and S30 were targeted by ERK and AMPK, respectively, we generated two GST-FoxO3A constructs, one encoding for the N-terminal domain [GST-FoxO3A(1–148)], and the other for a region encompassing residues 386–525 [GST-FoxO3A(386–525)] and containing serines previously shown to be targeted by both AMPK (S399 and S413)^12^ and ERK (S425)^30^ (Fig. 3i). Moreover, the GST-FoxO3A(1–148) vector was mutagenized by serine/alanine substitution at positions 12 and/or 30 to obtain the constructs GST-FoxO3A(1–148)S12A, GST-FoxO3A(1–148)S30A and GST-FoxO3A(1–148)S12A/S30A (Fig. 3i). The indicated purified proteins were subjected to a kinase assay to test the ability of AMPK and ERK to directly phosphorylate their candidate residues *in vitro*. Data presented in Fig. 3l show that ERK could efficiently phosphorylate GST-FoxO3A(1–148) as well as the positive control [GST-FoxO3A(386–525)], and that the S12A substitution was sufficient to significantly abrogate ^32^P. incorporation in GST-FoxO3A(1–148). Similar results were obtained with the S12A/S30A double-mutant, indicating that ERK directly phosphorylated FoxO3A at serine 12. To ascertain whether S30 could be phosphorylated by AMPK, we assayed GST-FoxO3A(1–148) and GST-FoxO3A(385–525) with AMPK and found that AMPK efficiently phosphorylated FoxO3A N-terminus * in vitro* (Fig. 3m). Serine-to-alanine substitution at position 30 abolished the radioactive labeling and similar results were obtained with the S12A/S30A double-mutant, thus indicating that AMPK phosphorylates FoxO3A at S30 (Fig. 3l).

Of note, S30 is more conserved across species than S12 (Supplementary Figure S4a) and is part of a highly conserved subdomain, which is shared by other human FoxOs, while S12 is not (Supplementary Figure S3).

### mtFoxO3A regulates the expression of the mitochondrial genome in metabolically stressed cancer cells

**Fig. 4:**
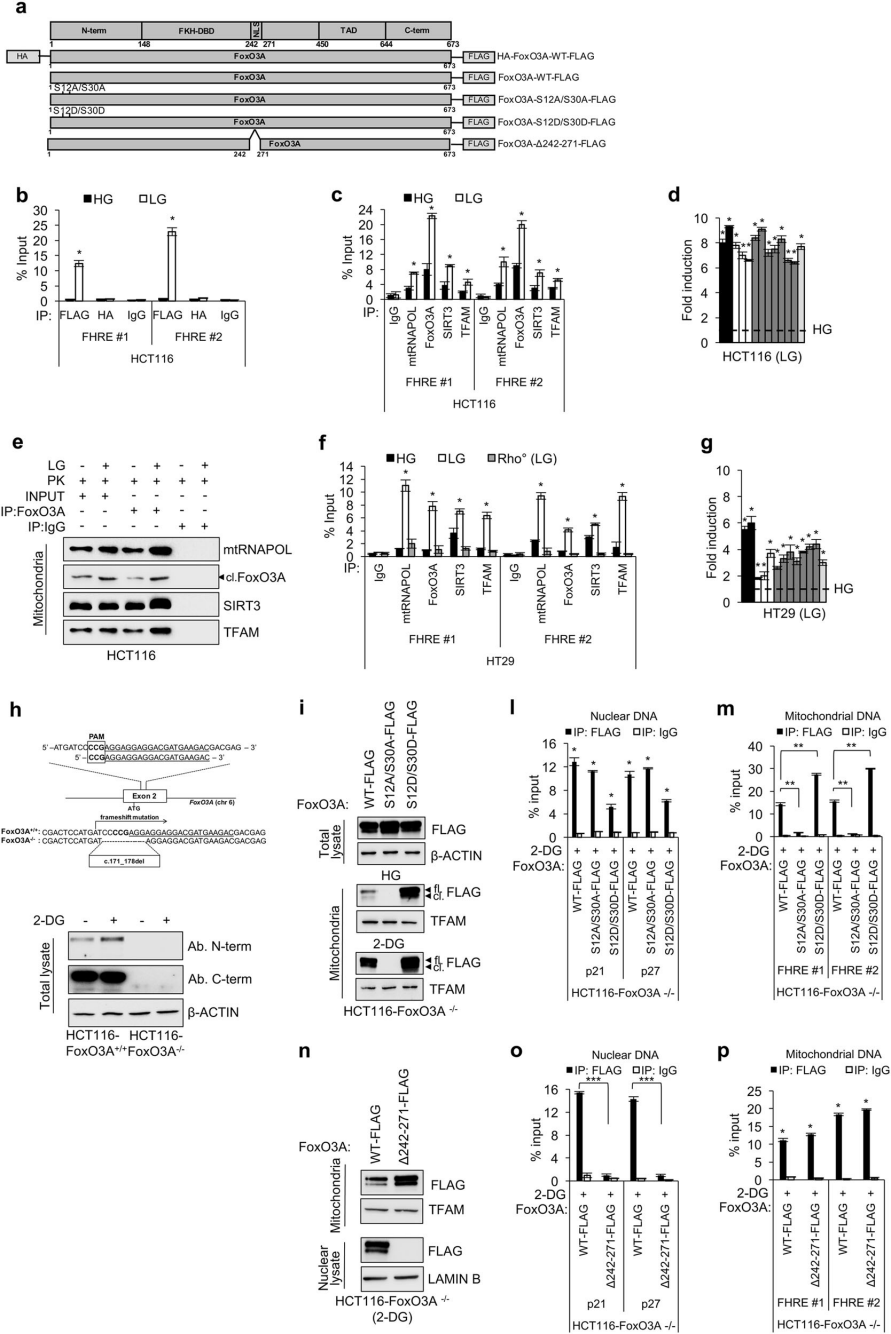
Mitochondrial FoxO3A regulates mitochondrial gene expression in metabolically stressed cancer cells. **a** Scheme of plasmids used. **b** ChIP analysis of exogenous FoxO3A recruitment at FHRE #1–2 sites on mtDNA (FHRE #1: bp 14,963–15,110; FHRE #2: bp 15,400–15,469) upon LG (0.75 mM glucose, 24 h) in HCT116 cells transfected with HA-FoxO3A-WT-FLAG. **c**,** f** ChIP analysis of endogenous FoxO3A recruitment at FHRE #1–2 sites on mtDNA in HCT116 (**c**), and HT29 and Rho^0^ HT29 (negative control) (**f**) cells upon LG (24 h). **d**,** g** Mitochondrial gene regulation in HCT116 (**d**) and HT29 (**g**) cells upon LG (24 h) assessed by RT-PCR. Black bars: *ATPase 6* and 8 genes; white bars: *COX1, COX2*, and *COX3* genes; gray bars: *ND1, ND2, ND3, ND4, ND4L, ND5*, and *ND6* genes; light gray bar: *CYTOCHROME B* gene. The dotted line corresponds to the expression levels detected in cells cultured in HG. **e** Co-immunoprecipitation analysis with the indicated antibodies of PK-treated HCT116 mitochondrial fractions upon LG (24 h). **h** Upper panel: scheme of gRNA location in human FoxO3A locus. Targeting sites and proto-spacer adjacent motifs (PAMs) and the deleted region are indicated. Lower panel: immunoblot analysis of HCT116-FoxO3A^+/+^ and HCT116-FoxO3A^−/−^ cells with different anti-FoxO3A antibodies upon 2-DG (1 mM, 6 h) treatment. **i**–**p** HCT116-FoxO3A^−/−^ cells were transfected with the indicated FoxO3A plasmids for 48 h and treated with 2-DG (1 mM, 6 h). **i** Immunoblot analysis of total and mitochondrial proteins. **l**,** m**,** o**,** p** ChIP analysis of exogenous FoxO3A recruitment at FHRE *p21–p27* sites on nuclear DNA (**l**,** o**) and FHRE #1–2 sites on mtDNA (**m**,** p**). **n** Immunoblot analysis of nuclear and mitochondrial proteins. **b**,** c**,** f**,** l**,** m**,** o**,** p** Anti-IgGs were used as controls. **e**,** h**,** i**,** n** β-actin, TFAM, and LAMIN B were used as total, mitochondrial and nuclear lysate controls, respectively, as appropriate. fl. full-length FoxO3A, cl. cleaved FoxO3A, N-term. N-terminal domain, FKH-DBD forkhead DNA-binding domain, NLS nuclear localization signal, TAD transactivation domain, C-term. C-terminal domain. The presented results are representative of at least three independent experiments. Where applicable, data are presented as mean ± SEM and significance was calculated with Student’s *t* test; *p < 0.05, **p < 0.01, and ***p < 0.001

To functionally characterize the newly identified FoxO3A cleaved form, we expressed the HA-FoxO3A-FLAG vector (Fig. 4a) in HCT116 cancer cells. Chromatin immunoprecipitation (ChIP) analysis performed using an anti-FLAG or an anti-HA antibody showed that the cleaved form, recognized only by the anti-FLAG antibody, could efficiently bind the D-loop of mtDNA at FHRE consensus sites under metabolic stress conditions (Fig. 4b). Further, ChIP analyses performed on endogenous proteins from mitochondria purified from different human cancer cell lines and HEK293 cells confirmed that FoxO3A-cl. is recruited at FHRE sites together with SIRT3, TFAM and mtRNAPOL in glucose-restricted cells (Fig. 4c, f and Supplementary Figure S6a, S6b), and that these proteins form a complex into the matrix (Fig. 4e). Specificity was ensured by a parallel analysis carried out in mitochondria purified from mtDNA-depleted (Rho^0^) HT29 cells^31^, which served as a negative control (Fig. 4f). Importantly, the presence of the FoxO3A-cl./SIRT3/TFAM/mtRNAPOL complex on mtDNA correlated well with increased expression of all mitochondrial transcripts in LG conditions, as shown by quantitative RT-PCR analysis of mtRNA (Fig. 4d, g and Supplementary Figure S6a, S6b).

To dissect FoxO3A nuclear vs. mitochondrial activity in cancer cells under metabolic stress, we first generated a FoxO3A knockout HCT116 cell line (HCT116-FoxO3A^−/−^) with the CRISPR/Cas9 system for genome editing (Fig. 4h). Then, we reconstituted FoxO3A expression by transfecting these cells with wild-type FoxO3A or the unphosphorylatable mutant FoxO3A–S12A/S30A or a newly generated mutant, FoxO3A-S12D/S30D, where serines were replaced with aspartic acid residues to mimic phosphoserines (Fig. 4a). As shown in Fig. 4i, the phoshomimetic mutant (FoxO3A-S12D/S30D) strongly accumulated into the mitochondria, also in the absence of stress (HG), where it bound mtDNA and induced the expression of mitochondrial-encoded proteins, while FoxO3A-S12A/S30A failed to do so (Fig. 4m and Supplementary Figure S6c, S6d). Importantly, both FoxO3A mutants were still able to enter the nucleus and bind FHRE sites at the promoters of *p21* and *p27* target genes under metabolic stress (Fig. 4l). In the same cellular system, another FoxO3A mutant, FoxO3A-Δ242-271, in which we deleted the nuclear localization signal (NLS)^32^, was unable to enter the nucleus (Fig. 4n) and bind *p21/p27* FHRE sites (Fig. 4o), but was still capable of entering the mitochondria and bind FHRE sites at the D-Loop of mtDNA in metabolically stressed cancer cells (Fig. 4p).

### FoxO3A accumulation into the mitochondria only requires the AMPK signal in normal cells and tissues under nutrient shortage

**Fig. 5:**
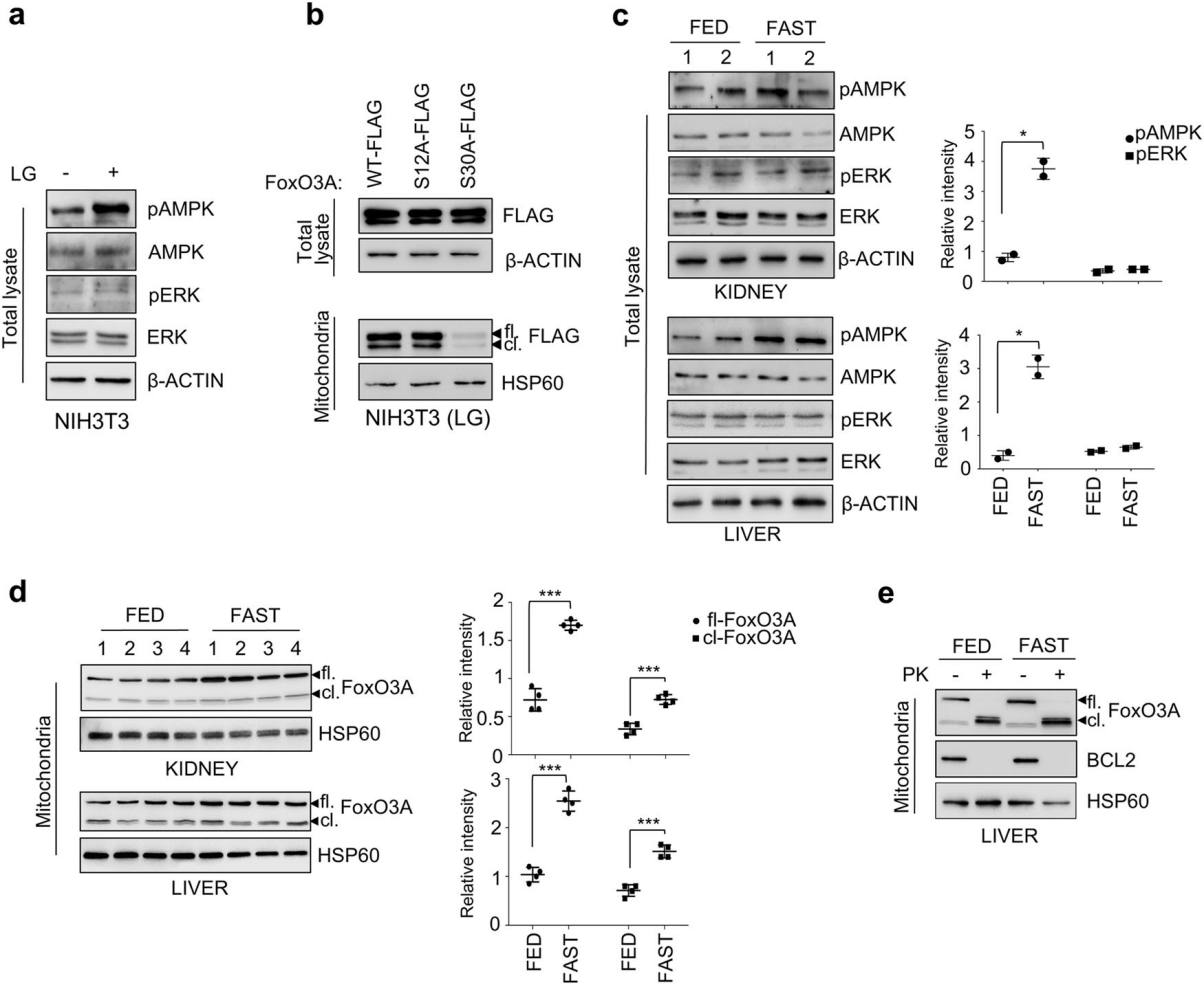
FoxO3A accumulation into the mitochondria only requires the AMPK signal in normal cells and tissues under nutrient shortage. **a** Immunoblot analysis of total proteins isolated from NIH3T3 cells upon LG (0.75 mM glucose, 24 h). β-actin: loading control. **b** Immunoblot analysis of total and mitochondrial proteins isolated from NIH3T3 cells transfected with the indicated plasmids for 48 h and subjected to LG (24 h). β-actin and HSP60 were used as total lysate and mitochondria controls, respectively. **c** Left panel: immunoblot analysis of total proteins isolated from kidney and liver of fed or fasted (18 h) mice. β-actin: loading control. Right panel: densitometric analysis of the phosphorylated forms of AMPK and ERK normalized against total AMPK and ERK, respectively, and the loading control. **d** Left panel: immunoblot analysis of mitochondrial proteins isolated from kidney and liver of fed or fasted (18 h) mice. HSP60: loading control. Right panel: densitometric analysis of full-length and cleaved FoxO3A normalized against the mitochondrial fractionation loading control. **e** Immunoblot analysis of mitochondrial proteins isolated from the liver of fed or fasted (18 h) mice in the presence or absence of PK. BCL2: outer membrane control, HSP60: mitochondrial matrix control. fl. full-length FoxO3A, cl. cleaved FoxO3A. The presented results are representative of at least three independent experiments. Where applicable, data are presented as mean ± SEM and significance was calculated with Student’s *t* test; *p < 0.05, **p < 0.01, and ***p < 0.001

To explore FoxO3A “mitochondrial code” in normal cells, we transfected NIH3T3 fibroblasts with FoxO3A-wt, FoxO3A-S12A or FoxO3A-S30A. Of note, normal fibroblasts only required AMPK activation to induce mtFoxO3A accumulation, as replacement of serine 30 with a non-phosphorylatable alanine was sufficient to prevent FoxO3A mitochondrial localization under glucose restriction (Fig. 5a, b).

Then, to corroborate our data *in vivo*, we subjected mice to overnight fasting (18 h), a procedure known to significantly lower plasma glucose levels^33^, and purified mitochondria from kidney and liver. Immunoblot analysis revealed the accumulation of both FoxO3A forms in fasted mice, suggesting that nutrient shortage caused localization of the transcription factor at the outer mitochondrial membrane (FoxO3A-fl.) and its subsequent import, cleavage, and translocation into the mitochondrial matrix (FoxO3A-cl.) (Fig. 5d, e). PK treatment of mouse mitochondria confirmed that the cleaved form is detectable also *in vivo* into the mitochondrial matrix (Fig. 5e). To get the full picture, we characterized the status of AMPK and ERK in kidney and liver of fasted mice (Fig. 5c). Only AMPK was found to be significantly activated in these animals upon nutrient shortage, which supports its role in the signal transduction pathways leading to FoxO3A mitochondrial translocation.

These results suggest that normal cells and tissues under metabolic stress only require the AMPK signal on S30 to direct FoxO3A into the mitochondria, whereas ERK involvement in FoxO3A mitochondrial localization is unique to tumor cells.

### Role of mtFoxO3A in cancer cell response to metabolic stress

**Fig. 6:**
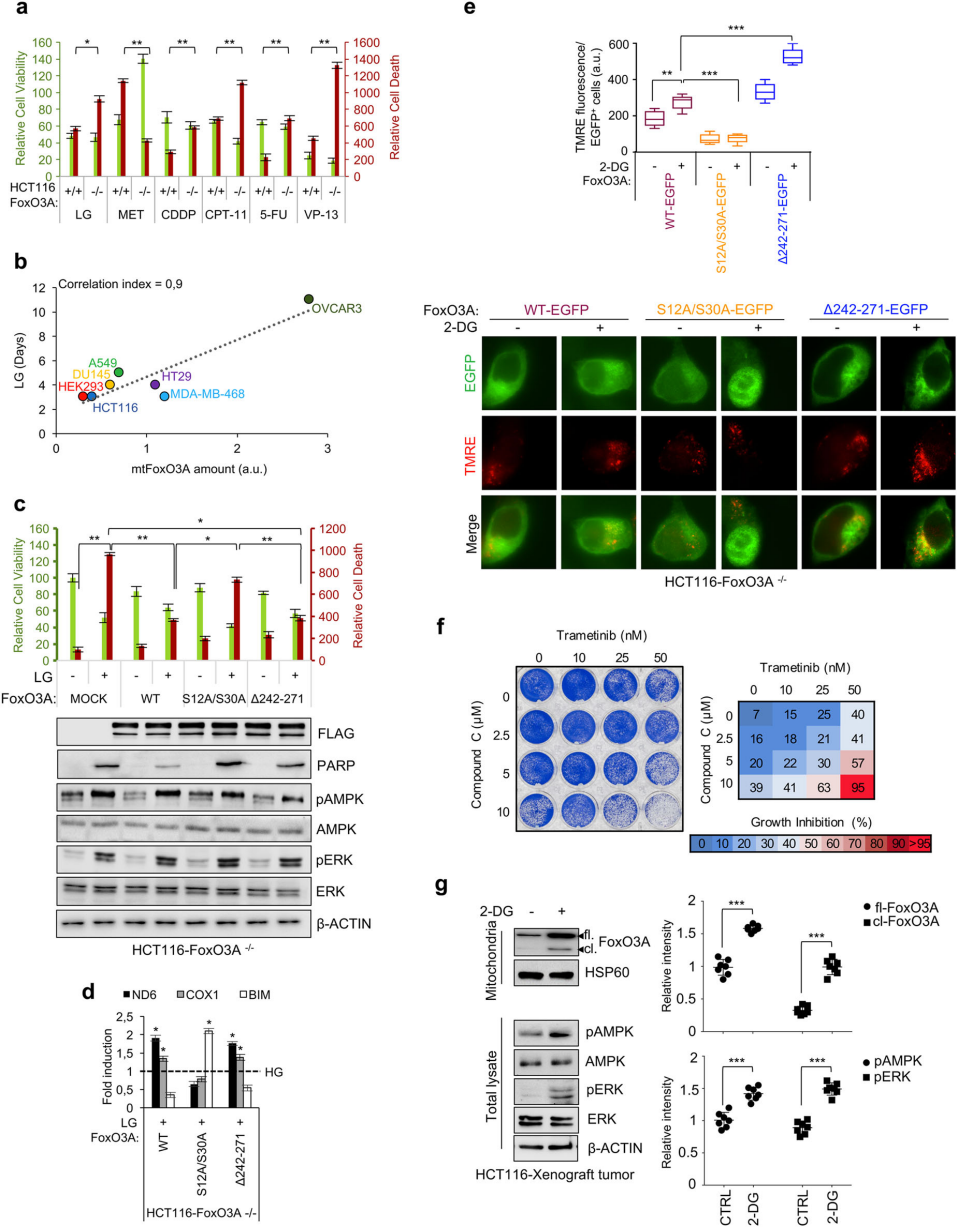
mtFoxO3A is involved in cancer cell response to metabolic stress. **a** HCT116-FoxO3A^+/+^ and HCT116-FoxO3A^−/−^ cells were subjected to different treatments: glucose restriction (LG, 0.75 mM glucose, 24 h), metformin (MET, 10 μM, 72 h), cisplatin (CDDP, 30 μM, 48 h), irinotecan (CPT-11, 30 μM, 24 h), 5-fluorouracil (5-FU, 2 μM, 24 h) and etoposide (VP-13, 40 μM, 24 h). Relative cell viability and relative cell death were calculated. **b** Correlation between LG-resistance (days) and mitochondrial FoxO3A (mtFoxO3A) protein levels in different human cell lines (HCT116 and HT29 colorectal cancer cells, HEK293 embryonic kidney cell, DU145 prostate cancer cells, A549 lung cancer cells, MDA-MB-468 breast cancer cells and OVCAR3 ovarian cancer cells). a.u. arbitrary units. **c** HCT116-FoxO3A^−/−^ cells were transfected with the indicated plasmids (48 h) and subjected to LG (24 h). Upper panel: relative cell viability and relative cell death. Lower panel: immunoblot analysis of total proteins. β-actin: loading control. **d** Transcription analysis of selected mitochondrial (*ND6* and *COX1*) and nuclear (*BIM*) genes by RT-PCR in HCT116-FoxO3A^−/−^ cells transfected with the indicated plasmids (48 h) and subjected to LG (24 h). **e** HCT116-FoxO3A^−/−^ cells, transfected with the indicated plasmids (48 h), were subjected to metabolic stress with 2-DG (1 mM, 6 h). The graph reflects the quantification of tetramethylrhodamine ethyl ester (TMRE) fluorescence of active mitochondria in transfected cells. **f** Clonogenic assay on HCT116-FoxO3A^+/+^ cells cultured in LG (24 h) and treated with increasing concentrations of trametinib and/or compound C, as indicated, for 24 h. Cell growth percent inhibition at each drug concentration is presented. **g** Left panel: immunoblot analysis of total and mitochondrial proteins isolated from tumors (*n* ≥ 7 for each group) derived from HCT116-xenografted nude mice subjected to 2-DG treatment (100 mg/kg, 6 days). β-actin and HSP60 were used as total and mitochondrial loading control, respectively. Right panel: densitometric analysis of full-length and cleaved FoxO3A normalized against the mitochondrial loading control and the results of the densitometric analysis of the phosphorylated-AMPK and ERK normalized against total AMPK and ERK, respectively, and the loading control. Data are presented as mean ± SEM and significance was calculated with Student’s *t* test; *p < 0.05, **p < 0.01, and ***p < 0.001

To evaluate the role of FoxO3A in tumor cells subjected to metabolic stress or treated with cancer therapeutics, HCT116-FoxO3A^+/+^ and HCT116-FoxO3A^−/−^ cells were cultured in LG conditions or in the presence of drugs currently administered to colorectal cancer patients and whose activity has been shown to involve FoxO3A in cellular models (metformin, cisplatin, irinotecan, 5-fluorouracil and etoposide)^8,34–37^. Surprisingly, HCT116-FoxO3A^−/−^ cells were more sensitive to metabolic stress and chemotherapeutics than their wild-type isogenic counterparts, suggesting that the presence of FoxO3A results in resistance and survival (Fig. 6a). Conversely, HCT116-FoxO3A^+/+^ cells were more sensitive to metformin treatment than HCT116-FoxO3A^−/−^ cells (Fig. 6a). Then, we cultured several human cancer cell lines and HEK293 cells in LG conditions to assay their resistance under metabolic stress. Our results showed that the higher the amount of mtFoxO3A they accumulated, the more they survived in LG cultures (Fig. 6b), suggesting that mtFoxO3A could represent a survival factor in these conditions. To test this hypothesis, we reconstituted FoxO3A expression in HCT116-FoxO3A^−/−^ cells by transfection with FLAG-tagged vectors encoding for FoxO3A-wt, the unphosphorylatable double-mutant FoxO3A-S12A/S30A (impaired in its mitochondrial localization, but still able to localize into the nucleus and bind target genes, see Fig. 4i–m) or FoxO3A-Δ242-271 (which lacks the NLS, but is able to enter the mitochondria and bind FHRE sites at mtDNA, see Fig. 4n–p) and cultured them in LG conditions. Our results showed that reconstitution of FoxO3A-wt expression results in increased survival; of note, FoxO3A-Δ242-271 was still able to rescue metabolic stress-dependent cell death, while the mutant impaired in mitochondrial localization failed to do so (Fig. 6c). Furthermore, analysis of FoxO3A target gene expression revealed that both the wild-type form and the Δ242-271 mutant were able to activate mitochondrial transcription in surviving cells, while the FoxO3A-S12A/S30A mutant contributed to apoptosis induction in metabolically stressed cancer cells by promoting *BIM* transcription (Fig. 6d). Subsequently, we transfected FoxO3A knockout cells with GFP-tagged vectors encoding for FoxO3A-wt, FoxO3A-S12A/S30A, or FoxO3A-Δ242-271, then we induced metabolic stress by adding 2-DG and performed TMRE staining to visualize the polarization status of mitochondrial membranes in GFP-positive cells. Our data indicate that mtFoxO3A is required to maintain the membrane potential of functionally active and healthy mitochondria in cancer cells subjected to metabolic stress (Fig. 6e). Indeed, TMRE failed to stain mitochondria of HCT116-FoxO3A^−/−^ cells transfected with the FoxO3A-S12A/S30A mutant (Fig. 6e), as they were undergoing apoptosis (Fig. 6c). These results highlighted the functional importance of FoxO3A S12 and S30 residues, together with the activation of pathways dictating their phosphorylation (Fig. 6c), in cancer cell resistance to metabolic stress, and prompted us to evaluate the inhibition of the MEK/ERK and/or the AMPK pathway in HCT116-FoxO3A^+/+^ cells cultured in LG conditions. Of note, trametinib (a MEK inhibitor approved by the FDA for clinical use) and CC (an AMPK inhibitor) showed a synergistic cytotoxic effect in metabolically stressed cancer cells (Fig. 6f). In order to confirm these results *in vivo*, we injected HCT116-FoxO3A^+/+^ cells in nude mice and treated growing tumors with 2-DG to induce metabolic stress. Our results reveal that activation of both the MEK/ERK and the AMPK pathways is induced in tumor tissues upon metabolic stress, together with FoxO3A mitochondrial localization, similar to what happens in culture (Fig. 6g).

### Role of mtFoxO3A in cancer cell response to chemotherapeutic agents

**Fig. 7:**
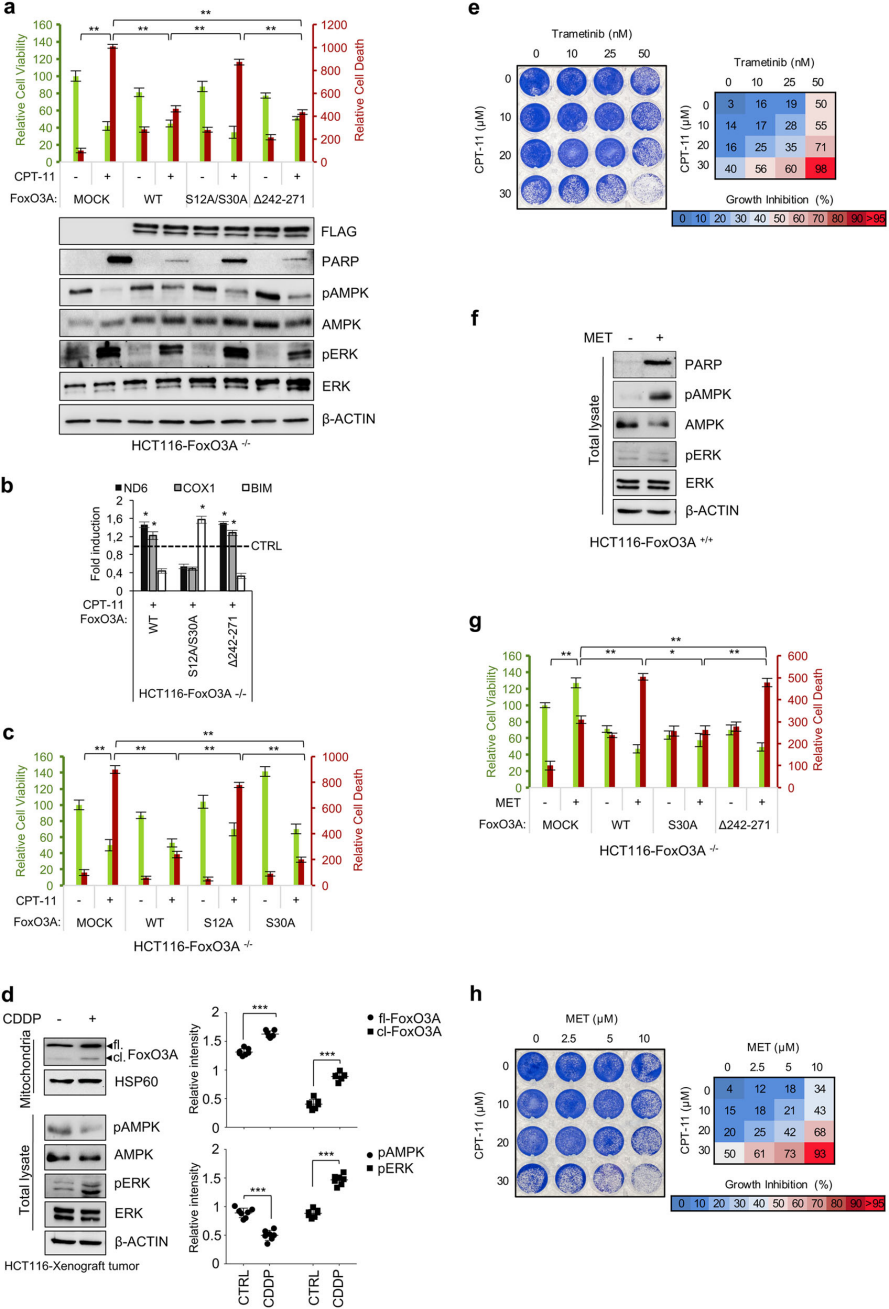
mtFoxO3A is involved in cancer cell response to chemotherapeutic agents. **a**–**c** HCT116-FoxO3A^−/−^ cells were transfected with the indicated plasmids for 48 h and then treated with irinotecan (CPT-11, 30 μM, 24 h). **a** Upper panel: relative cell viability and relative cell death. Lower panel: immunoblot analysis of total proteins. β-actin: loading control. **b** Transcription analysis of selected mitochondrial (*ND6* and *COX1*) and nuclear (*BIM*) genes by RT-PCR. **c** HCT116-FoxO3A^−/−^ cells were transfected with the indicated plasmids for 48 h and then treated with irinotecan (CPT-11, 30 μM, 24 h). Relative cell viability and relative cell death were calculated. **d** Left panel: immunoblot analysis of total and mitochondrial proteins isolated from tumors (*n* ≥ 7 for each group) derived from HCT116-xenografted nude mice subjected to cisplatin treatment (CDDP, 2 mg/kg, 6 days). β-actin and HSP60 were used as total lysate and mitochondrial fraction controls, respectively. Right panel: densitometric analysis of full-length and cleaved FoxO3A normalized against the mitochondrial fractionation loading control and the results of the densitometric analysis of the phosphorylated forms of AMPK and ERK normalized against total AMPK and ERK, respectively, and the loading control. **e** Clonogenic assay on HCT116-FoxO3A^+/+^ cells treated with increasing concentrations of trametinib (24 h) and/or irinotecan (24 h), as indicated. **f** Immunoblot analysis of total proteins isolated from HCT116-FoxO3A^+/+^ cells upon metformin treatment (MET, 10 μM, 72 h). β-actin: loading control. **g** HCT116-FoxO3A^−/−^ cells were transfected with the indicated plasmids for 48 h and then treated with metformin (MET, 10 μM, 72 h). Relative cell viability and relative cell death were calculated. **h** Clonogenic assay on HCT116-FoxO3A^+/+^ cells treated with increasing concentrations of metformin (24 h) and/or irinotecan (24 h), as indicated. **e**,** h** Cell growth percent inhibition at each drug concentration is presented. The data presented are the mean of at least three independent experiments. Where applicable, data are presented as mean ± SEM and significance was calculated with Student’s *t* test; *p < 0.05, **p < 0.01, and ***p < 0.001

To investigate the role of mitochondrial vs. nuclear FoxO3A in cancer cell response to chemotherapeutic agents, HCT116-FoxO3A^−/−^ cells were transfected with FLAG-tagged vectors encoding for FoxO3A-wt, FoxO3A-S12A/S30A or FoxO3A-Δ242-271 and then cultured in the absence or presence of irinotecan (CPT-11), cisplatin (CDDP), 5-fluorouracil (5-FU) or etoposide (VP-13). Consistent with the data obtained in HCT116-FoxO3A^−/−^ vs. HCT116-FoxO3A^+/+^ cells (Fig. 6a), reconstitution of wild-type FoxO3A expression resulted in increased survival (Fig. 7a and Supplementary Figure S7a–c). Moreover, the mitochondrial localization-impaired unphosphorylatable mutant was unable to rescue chemotherapy-induced apoptosis (Fig. 7a and Supplementary Figure S7a–c), while it could activate *BIM* transcription to contribute to the apoptotic response (Fig. 7a, b), in line with our results in metabolically stressed cells. The FoxO3A-Δ242-271 mutant behaved similar to FoxO3A-wt and both were able to activate mitochondrial gene expression in chemotherapy-induced cellular stress conditions (Fig. 7a, b and Supplementary Figure S7a–c). Molecular analysis of transfected HCT116-FoxO3A^−/−^ cells showed activation of the MEK/ERK, but not the AMPK pathway, in response to chemotherapy (Fig. 7a, lower panel). Indeed, only serine 12 of FoxO3A was required to elicit chemoresistance in these conditions (Fig. 7c). Consistently, in HCT116-FoxO3A^+/+^ xenografted tumors treated with CDDP, we detected activation of the MEK/ERK, but not the AMPK pathway, and accumulation of FoxO3A into the mitochondria (Fig. 7d). Of note, the MEK inhibitor trametinib and CPT-11 showed a synergistic cytotoxic effect in HCT116-FoxO3A^+/+^ cells (Fig. 7e).

An opposite behavior was observed when HCT116-FoxO3A^+/+^ and HCT116-FoxO3A^−/−^ cells were treated with metformin. Indeed, molecular analysis of HCT116-FoxO3A^+/+^ cells indicated that metformin only activates AMPK and induces cells to undergo apoptosis (Fig. 7f). Reconstitution of FoxO3A expression with the wild-type form or the FoxO3A-Δ242-271 mutant significantly increased cell death (Fig. 7g), suggesting that mtFoxO3A is required for apoptosis induction by metformin. Indeed, reconstitution with a vector encoding for the FoxO3A-S30A mutant was sufficient to reduce cell death to mock-transfected cell levels. These results indicate that metformin can induce apoptosis via the AMPK-mtFoxO3A axis. Consistently, metformin showed a synergistic cytotoxic effect with irinotecan in HCT116-FoxO3A^+/+^ cells (Fig. 7h).

## Discussion

**Fig. 8:**
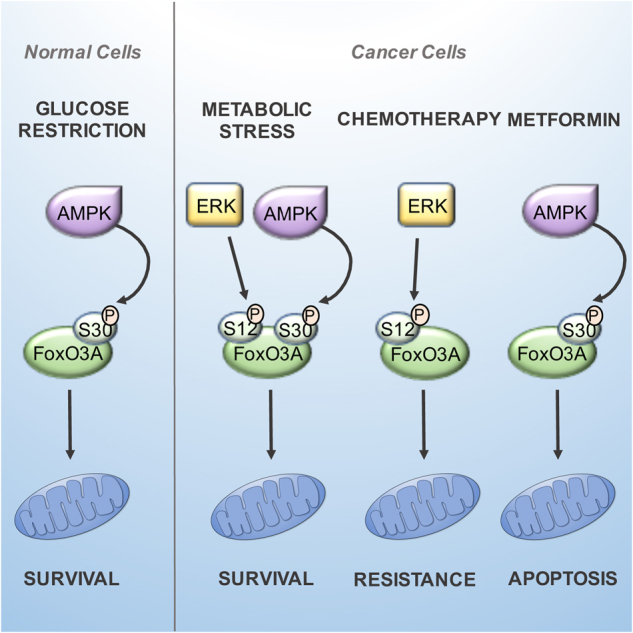
FoxO3A represents a survival factor in metabolically stressed cancer cells. Normal cells and tissues under metabolic stress require only the AMPK signal on S30 to direct FoxO3A into the mitochondria. It seems that ERK involvement in FoxO3A mitochondrial localization is exclusive to tumor cells, which reveals a critical difference between normal and cancer cells that could be exploited for cancer therapeutics. In metabolically stressed cancer cells, FoxO3A is recruited to the mitochondria through activation of MEK/ERK and AMPK, which phosphorylate serine 12 and 30, respectively, on FoxO3A N-terminal domain. Subsequently, FoxO3A is imported and cleaved to reach mitochondrial DNA, where it activates expression of the mitochondrial genome to support mitochondrial metabolism. In cancer cells treated with chemotherapeutic agents, accumulation of FoxO3A into the mitochondria promoted survival in a MEK/ERK-dependent manner, while mitochondrial FoxO3A was required for apoptosis induction by metformin

Recent advances in cancer understanding suggest that the “Warburg effect” should be reconsidered in its interpretation of the role of mitochondria in tumorigenesis. Indeed, although aberrant tumor cell growth is frequently associated with alterations of biochemical metabolism, mitochondrial function is not usually impaired^2–4^. In fact, large studies conducted to examine the mutational status of the mitochondrial genome in tumors revealed that detrimental and pathogenic mtDNA mutations are negatively selected^7,38,39^, implying that selective pressure for mitochondrial genome function preservation is a common feature in human tumors^5–7,40^. In addition to respiration, mitochondria are the powerhouse for bioenergetics and biosynthetic pathways that are required for tumorigenesis^7^. Thus, understanding the role of mitochondrial function in cancer might reveal novel approaches to targeted cancer therapy. Indeed, recent reports suggested that cancer cells are highly susceptible to the inhibition of oxidative phosphorylation, and that mitochondrial translation inhibitors can act as sensitizers to cancer therapeutics and chemotherapy^41,42^. Consistently, our results indicated that MEK inhibition by trametinib synergized with AMPK inhibitors in reducing survival under metabolic stress, and with irinotecan in inducing cytotoxicity in colorectal cancer cells (Fig. 8). Importantly, combination therapy with MEK inhibitors and chemotherapeutic agents is predicted to overcome resistance mechanisms and potentiate the antitumor activity of each single agent, and various phase II/III trials based on such approaches are currently ongoing^43^.

Metformin is a biguanide drug commonly used in diabetes treatment that has shown anticancer activity in diabetic patients^44,45^. It has been recently proposed that metformin inhibits the activity of complex I of the mitochondrial machinery responsible for oxidative phosphorylation, the same mechanism inducing AMPK activation in cultured cells^46,47^. In our cellular model, metformin activity was mediated by AMPK and required mtFoxO3A to elicit its pro-apoptotic effect in cancer cells and chemosensitization to irinotecan (Fig. 8). Importantly, several phase II/III clinical trials are currently evaluating the efficacy of metformin in association with chemotherapeutic agents as well as its chemoprevention activity as a single agent^44,45^.

The interplay between the MEK/ERK and the AMPK cascades, which converge on the N-terminal domain of FoxO3A, represents the first chapter of the mitochondrial tale of the FoxO3A code. Further studies are needed to establish whether other signaling pathways actually target FoxO3A N-terminus to modulate its mitochondrial localization and function and which stimuli do they respond to. This will be instrumental to devise personalized therapeutic strategies -employing molecularly targeted drugs- aimed at manipulating cellular metabolism to counteract cancer initiation and progression.

## Material and methods

### Cells culture and reagents

NIH3T3, MEFs, HT29, HT29 Rho^0^, HCT116, HCT116-FoxO3A^−/−^, HeLa, LS174T, MDA-MB-468, OVCAR3, A549, and SW-480 cells were cultured in DMEM high glucose (HG), without pyruvate (#11360-070, Gibco, Carlsbad, California) with 10% FBS (#0270-106, Gibco) and 100 IU/ml penicillin–streptomycin (#15140-122, Gibco). BT474, EFM19 and Caco2 cells were maintained in the same conditions with 5% and 20% FBS, respectively. HEK293 cells were supplemented with 1% pyruvate and NEAA (#11140, Sigma Aldrich, ST. Louis, Missouri). DU145 cells were cultured in RPMI high glucose (HG), without pyruvate (#21875-034, Gibco) with 10% FBS (Gibco) and 100 IU/ml penicillin–streptomycin (Gibco). All cell lines were glucose-restricted by using DMEM 0.75 mM (LG) as previously described^20^. Compound C (5 μM, #171260), AICAR (5 mM, #A9978), PD98059 (0,02 mM, #P215), 2-deoxy-glucose (1 mM, #D8375), iodoacetic acid (0,5 μM, #I4386), metformin (10 μM, #D150959), 5-fluorouracil (2 μM, #F6627), cisplatin (30 μM, #P4394), etoposide (40 μM, #E1383) and irinotecan (30 μM, #347609) were all from Sigma; trametinib (#S2673) was from Selleckchem (Munich, German).

### Animal care and use

C57/Bl6J male pure strain mice were bred on a 12 h light/dark cycle and fed with standard diet. Eight- to ten-week-old mice were divided in two experimental groups: free food access group (fed) and 18-h fasted group. Mice were killed at the beginning of the light cycle in order to avoid circadian variation of the gene expression profile. Brain, kidney, lung, pancreas, colon, and liver were chopped into small pieces with a razor blade and then disaggregated using a Medimachine (Becton Dickinson, NJ, USA). Cells were collected and processed for total protein analysis and/or mitochondria isolation as previously described^48^.

Female CD-1 athymic nude mice (6–8-week-old) were obtained from Charles River Laboratories (Wilmington, MA). To develop xenograft tumors, 10 × 10^6^ HCT116 cells were injected subcutaneously into the flanks (0.2 ml per flank) of CD-1 mice. The volume of the tumors was measured every 2–3 days and calculated using the following formula: volume (mm^3^) = (width)^2^ × length × 0.5. When the tumor volume reached 60 mm^3^, mice were randomized into different treatment groups. Cisplatin (2 mg/kg) or 2-deoxygluocose (100 mg/kg) were given intraperitoneally once every 3 days. Control groups received the vehicle only (normal saline). Mice were treated for 6 days and tumor volume and body weight were recorded every 2–3 days. At the end of the treatment, mice were killed and tumors explanted for immunoblot analysis. Animals were cared for following the institutional guidelines, in compliance with national and international laws and policies.

### Cloning

The plasmids described in the manuscript were obtained, with specific primers, as previously described^49^. Site-directed mutagenesis was performed using the Q5® Site-Directed Mutagenesis Kit (#E05545, New England Biolabs, Ipswich, MA) according to the manufacturer’s instructions. The p3xFLAG-CMV14-FoxO3A construct was obtained starting from the pECE-HA-FoxO3A plasmid (kindly provided by Dr. M. Greenberg, Addgene, Cambridge, USA, plasmid #1787); the p3xFLAG-CMV14-HA-FoxO3A construct was obtained starting from the pECE-HA-FoxO3A plasmid, followed by HA-FoxO3A fragment insertion into p3xFLAG-CMV14-backbone. FoxO3A-Δ1–148-FLAG, FoxO3A-S12A-FLAG, FoxO3A-S26A-FLAG, FoxO3A-S30A-FLAG, FoxO3A-S43A-FLAG, FoxO3A-S48A-FLAG, FoxO3A-S55A-FLAG, FoxO3A-S12A/S30A-FLAG, FoxO3A-S12D/S30D-FLAG, FoxO3A-Δ1-30-FLAG, FoxO3A-Δ80-108-FLAG and FoxO3A-Δ242-271-FLAG constructs were obtained starting from the p3xFlag-FoxO3A plasmid. FoxO3A(1–148)-GST and FoxO3A(386–525)-GST constructs were obtained starting from the p3xFLAG-FoxO3A plasmid, followed by FoxO3A(1–148) and FoxO3A(386–525)-GST insertion into a pGEX-4T-3 empty vector. FoxO3A(1–148)S12A-GST, FoxO3A(1–148)S30A-GST, FoxO3A(1–148)S12A/S30A-GST constructs were obtained starting from the pGEX-4T-3-FoxO3A(1–148) plasmid. The FoxO3A-WT-EGFP construct was obtained starting from the p3xFlag-FoxO3A plasmid, followed by FoxO3A-WT insertion into a pEGFP-N3 empty vector. FoxO3A–S12A/S30A-EGFP and FoxO3A–Δ242-271-EGFP constructs were obtained starting from the FoxO3A-WT-EGFP plasmid. Site-directed mutagenesis primer sequences used in this manuscript are available from the authors upon request.

### Cell transfection and RNA interference

HCT116 and HEK293 cells were transiently transfected with mammalian expression plasmids using TransIT-LT1 Transfection Reagent (#2300, Mirus, Madison, USA) according to the manufacturer’s instruction.

For RNA interference, HCT116 cells were transfected with 50 nM validated Silencer® Select Pre-Designed siRNA (Thermo Fisher Scientific, Waltham, MA) directed against FoxO3A by using the HiPerfect reagent (#301704, QIAGEN, Hilden, Germany) according to the manufacturer’s instructions. On-TARGET-plus control siRNAs (Thermo Fisher Scientific) were used as a non-silencing control. siRNA sequences used in this study:

siRNA1: 5′-CUCACUUCGGACUCACUUAtt-3′, 5′-UAAGUGAGUCCGAAGUGAGca-3′

siRNA2: 5′-GCCUUGUCGAAUUCUGUCAtt-3′, 5′-UGACAGAAUUCGACAAGGCac-3′

### Protein expression and purification

NEB5-alpha bacterial strains (#C2987I, New England Biolabs) transformed with different pGEX-4T3-GST-FoxO3A constructs were grown in Luria Broth medium with Ampicillin (#13399, Sigma) and induced with 0.5 mM IPTG when they reached the optical density of 0.6 (A600) at 37 °C, for 4 h. Cells were then collected by centrifugation and pellets were lysed with B-PER lysis buffer (#78248, Thermo Fisher Scientific). The lysate was centrifuged at 15,000 × *g* for 5 min at 4 °C. Recombinant protein expression was confirmed by SDS-PAGE. Fusion proteins were purified by affinity chromatography using the GST Bulk Kit (#27-4570-01, GE Healthcare, Milwaukee, WI) according to the manufacturer’s instructions. GST-fused proteins were evaluated and quantified by SDS-PAGE.

### Mitochondria isolation and treatment

Mitochondria-enriched fractions were obtained as described^48^. Briefly, cells were collected and centrifuged at 600 × *g* at 4 °C for 10 min. Then, the pelleted cells were resuspended in ice-cold IBc buffer (0.1 M Tris-MOPS, 0.1 M EGTA/Tris, 1 M sucrose) and homogenized using a Teflon-glass pestle. The homogenate was centrifuged at 600 × *g* for 10 min at 4 °C and the supernatant was collected and centrifuged at 7000 × *g* for 10 min at 4 °C. The pellet was resuspended in with ice-cold IBc and finally centrifuged at 7000 × *g* for 10 min at 4 °C. The obtained mitochondria were treated with proteinase K 4 U/ml (#17916, Ambion, Austin, TX) and/or Triton X-100 (0.25%) and analyzed by immunoblotting, as previously described^20^.

### Proteinase K protection assay

Mitochondrial fractions were resuspended in an isotonic buffer^50^ and incubated with various concentrations of proteinase K for 20 min on ice. Digestion was stopped with 2 mM phenylmethylsulfonyl fluoride (PMSF). Mitochondrial proteins were isolated, separated by SDS/PAGE and then detected by western blot analysis.

### Mitochondrial swelling experiments

Mitochondrial fractions were resuspended in an hypotonic buffer (10 mM MOPS-KOH) and incubated on ice for 15 min^50^. Half of the sample was then treated with proteinase K (25 µg/ml) on ice for 20 min. An aliquot of 2 mM PMSF was added to inactivate protease activity. Mitochondria and mitoplasts were recentrifuged and analyzed by SDS-PAGE.

### Nuclear extraction

Nuclear extraction was performed using Nuclear Extraction Kit according to the manufacturer’s instructions (#ab113474, Abcam, Cambridge, MA).

### Chromatin immunoprecipitation (ChIP)

For mtDNA chromatin immunoprecipitaion to eliminate genomic DNA contamination, mitochondrial fractions were treated with DNase I (4 U/100 mg wet mitochondria) (#AM2224, Ambion) for 10 min on ice in the presence of 2.5 mM MgCl_2_ in mitochondria isolation buffer^48^. The reaction was stopped by adding EDTA (final concentration 50 mM) into the mixture and crude mitochondria were washed once. ChIP analysis was performed as previously described^20^. Briefly, formaldehyde (#F8775, Sigma) was added directly to mitochondrial fraction to a final concentration of 1%. Cross-linking was allowed to proceed for 15 min at room temperature and then stopped by the addition of glycine (#G8898, Sigma) to a final concentration of 0.125 M. Cross-linked mitochondria were lysed in CLB (10 mM Tris pH 8.0, 10 mM NaCl, 0.2% NP40) plus protease inhibitors (#04693124001, Roche, Mannheim, Germany). The mitochondrial fraction was enriched by differential microcentrifugation. The chromatin solution was sonicated, cleared by centrifugation, and the supernatant was divided into aliquots. One percent of the supernatant was taken as input. Four micrograms of antibody per point were used to immunoprecipitate chromatin-bound complexes. Immunoprecipitation was performed on a rotating platform overnight at 4 °C with the indicated antibodies. IgG were used as unrelated antibodies. Immunocomplexes were pulled down using protein G (#10003D, GE Healthcare). Following extensive washing, bound DNA was reverse cross-linked, purified using phenol:chloroform (#AM9720, Sigma) and analyzed by quantitative real-time PCR. ChIP assays on nuclear DNA were performed using the MAGnify chromatin immunoprecipitation system (#A11250, Life Technologies, Waltham, MA) according to the manufacturer’s instructions. IgG antibodies were included in the kit and 1 μg of indicated antibodies were used for each assay. Primer sequences used in this manuscript are available from the authors upon request.

### *In vitro* kinase assays

Purified AMPK (#171536, EMD Millipore, Billerica, MA) and/or ERK (#31152, Active Motif, Carlsbad, CA) were incubated with various recombinant substrates (0.5 μg) in kinase reaction buffer [HEPES (32 mM) pH 7.4, dithiothreitol (650 μM), Mg(CH_3_COO)_2_ (10 mM), BriJ-35 (0,012%), cold ATP (50 μM)] and 0.9–1.8 μCi of [γ-32P]ATP (Perkin Elmer, Waltham, MA) at 30 °C for 30 min. For AMPK, AMP (100 μM) was added to the reaction. Phosphorylation was detected by incorporation of radiolabeled [γ-32P]ATP.

### Immunoblotting

Protein extracts were obtained by treating cells or mitochondrial fractions with total lysis buffer (50 mM Tris-HCl pH 7.4, 250 mM NaCl, 5 mM EDTA, 0,1% Triton X-100, 1 mM DTT, 1 mM PMSF) supplemented with protease and phosphatase inhibitors (Roche). Total of 15–20 mg of protein extracts from each sample were denatured in 5× Laemmli sample buffer and loaded into an SDS–poly-acrylamide gel for western blots analysis. FoxO3A (CST #BK99199S), FoxO3A (CST #2497), COX4 3E11 (CST #4850), BCL2 50E3 (CST #2870), PDH C54G1 (CST #3205), FLAG M2 (Sigma #F1804), HA-Tag C29F4 (CST #3724), β-actin (CST #3700), AMPK-α Thr 172 (CST #2531), AMPK-α (CST #2532), p44/42 MAP2K1-ERK1/2 Thr202/Tyr204 (CST #9106), p44/42 MA2PK1-ERK1/2 (CST #9102), p-Acetyl-CoA Ser 79 (CST #3661), TFAM D5C8 (CST #8076), HSP60 D307 (CST #4870), IgG (CST #2729S), mtRNAPOL H300 (Santa Cruz #SC-67350), SIRT3 D22A3 (CST #5490), LAMIN B1 (CST #12586S) (Cell Signaling Technologies, Danvers, MA) PARP1 (p85 fragment, G7341, Promega, Madison, WI), ATPase 6 (#PA5-37129, Thermo Fisher Scientific), COXI (#A6403, Molecular probes, Carlsbad, CA), ND6 (#A31857, Molecular probes) were used as primary antibodies. HRPO-conjugated antibodies (#NA934V, #NA931V, GE Healthcare) were used as secondary antibodies and revealed using the ECL-plus chemiluminescence reagent (GE Healthcare). The densitometric evaluation was performed by ImageJ software.

### Microscopic quantification of viability and cell death

Cell viability and cell death of the reported cell lines were scored by counting, as previously described^34^. The supernatants (containing dead/floating cells) were collected, and the remaining adherent cells were detached by Trypsin/EDTA (#15400-054, Sigma). Cell pellets were resuspended in 1× PBS and 10 μl were mixed with an equal volume of 0.01% trypan blue solution. Viable cells (unstained, trypan blue negative cells) and dead cells (stained, trypan blue positive cells) were counted with a phase contrast microscope. The percentages of viable and dead cells were calculated.

### Immunogold labeling

The immunogold labeling assay was performed as previously described^20^. Briefly, cells were fixed with 4% formaldehyde and 0.005% glutaraldehyde, washed, incubated with the primary antibody overnight, and then with nanogold-conjugated Fab fragments of the secondary antibodies (Nanoprobes, New York, USA) for 2 h. Nanogold particles were developed using the gold-enhance kit (Nanoprobes). For each experimental condition, 20 cells were analyzed. In each cell, 15 areas (mean 3 × 106 nm^2^) were randomly selected. Data presented in the “Results” section were obtained by scoring the percentage of FoxO3A-positive cells and counting the number of gold particles per single mitochondria.

### Real-time PCR

RNA extraction and real-time PCR were performed as previously described^51^. Briefly, total RNA was extracted with Trizol reagent (#15596-018, Invitrogen, Carlsbad, CA) following the manufacturer’s instructions. Samples were then treated with DNase-1 (#AM2224, Ambion) and 4 μg of total RNA was retrotranscribed using iScript™ cDNA Synthesis Kit (#1708891, BIORAD, Hercules, CA) following the manufacturer’s instructions. PCRs were carried out in triplicate using the SYBR Green PCR Master Mix (#172-5275, BIORAD) on a QuantStudio3 Real-Time PCR System (Applied Biosystem, Carlsbad, CA), according to the manufacturer’s instructions. Relative quantification was done using the ΔΔCt method. Primer sequences used in this manuscript are available from the authors upon request.

### Co-immunoprecipitation (Co-IP)

For co-IP, cells (mitochondria) were lysed in IP lysis buffer (50 mM Tris-HCl pH 7.4, 250 mM NaCl, 5 mM EDTA, 0,1% Triton X-100) supplemented with protease inhibitors (Roche). Lysates were cleared by centrifugation and incubated overnight at 4 °C with 1 μg of anti-FLAG, anti-FoxO3A and/or IgG covalently bound to Protein G-Sepharose (#10003D, GE Healtcare)/Protein A-Sepharose (#17-0780-01, GE Healtcare). Immunocomplexes were washed twice with lysis buffer, boiled in Laemmli sample buffer and subjected to SDS-PAGE and western blot analysis.

### Prediction analysis

Analysis of the N-terminal region of FoxO3A (corresponding to positions 1–148) was performed using the NetPhos algorithm (http://www.cbs.dtu.dk/services/NetPhos) and Disphos prediction (Disorder-Enhanced Phosphorylation Sites Predictor, http://www.dAbi.temple.edu/disphos/). The KinasePhos 2.0 (http://kinasephos2.mbc.nctu.edu.tw/) prediction server was employed to search for consensus phosphorylation motifs. A comparative analysis between AMPK consensus phosphorylation motifs (UniProtKB/Swiss-Prot, http://www.uniprot.org/) and the N-terminal region of FoxO3A (corresponding to positions 1–148) was performed. MPP and MIP consensus motif-like peptide probes were used to search exact matches in the N-terminal region of FoxO3A (corresponding to positions 1–148).

### Colony formation assay

Colony formation assays were performed as previously described^34^. Briefly, cells were cultured in 60 mm dishes in the presence or absence of indicated drugs. After 24 h, media were discarded and cells were washed twice with 1× PBS. An aliquot of 2 ml of Coomassie brilliant blue (#161-0400, BIORAD) were added into each dish for 5 min and then cells were washed with ethanol 70% to remove the excess of Coomassie. Plates were dried at room temperature. Percent cell growth inhibition at each concentration was quantified by densitometric evaluation using ImageJ software.

### Mitochondrial membrane potential assay (TMRE staining) and immunofluorescence

Mitochondrial membrane potential assay was performed using tetramethylrhodamine ethyl ester perchlorate (TMRE) (#87917, Sigma), according to the manufacturer’s instructions. Nuclei were counterstained using DAPI (#D1306, Invitrogen). HCT116-FoxO3A^−/−^ cells were transiently transfected with eGFP-tagged mammalian expression plasmids (auto-fluorescence). After transfection and treatment, cells were fixed with 4% paraformaldehyde, permeabilized with 1% Triton X-100, and then stained as described above. Images were acquired using an Axio Observer Z1 microscope (Zeiss, Jena, Germany) and quantification of mitochondrial TMRE fluorescence in GFP^+^ cells was performed using ZEN imaging software (Zeiss).

### CRISPR/Cas9 system

The CRISPR/Cas9 reporter vector (GeneArt CRISPR Nuclease Vector Kit, #A21175, Invitrogen) was used according to the manufacturer’s instructions. HCT116 cells were transfected using Lipofectamine 3000 (#L3000015, Thermo Fisher Scientific) according to the manufacturer’s instruction. Isolation of clonal populations was performed with agarose-based cloning rings (#C1059, Sigma). Cell clones were tested for site-specific deletions by PCR. Sequencing products were purified using the Dye Ex 2.0 Spin Kit (#63204, QIAGEN, Germantown, MD) and sequenced on an ABI PRISM 310 Genetic Analyzer (Applied Biosystems).  Oligonucleotide sequences used in this study to create the gRNA vector:

gRNA top strand oligo: GTCTTCATCGTCCTCCTCCT

gRNA bottom strand oligo: AGGAGGAGGACGATGAAGAC

### Glucose restriction resistance assay

Glucose restriction resistance assays were performed on several cell lines. For each of them, a preliminary experiment was performed to determine the glucose restriction end point. Total of 2.5 × 10^4^ cells were plated for each cell line on a 12-well dish and switched 24 h later to low glucose medium (0.75 mM glucose). Every 24 h, the supernatants (containing dead/floating cells) were collected, and the remaining adherent cells were detached by Trypsin/EDTA (Sigma). Cell pellets were resuspended in 1× PBS and 10 μl were mixed with an equal volume of 0.01% trypan blue solution. Viable cells (unstained, trypan blue negative cells) and dead cells (stained, trypan blue positive cells) were counted with a phase contrast microscope. For each cell line, the glucose restriction end point was established as the time point in which the amount of dead cells was at least 50% of the total. To investigate the correlation between glucose restriction resistance and FoxO3A protein levels, 2 × 10^5^ cells were plated on 60-mm dishes and switched 24 h later to low glucose medium (0.75 mM glucose). For each cell line, cells were collected at the glucose restriction end point preliminarily established as described above. Then, cell pellets were lysed and total proteins were analyzed by immunoblot with a FoxO3A antibody (CST). Densitometric evaluation of FoxO3A protein levels was performed by ImageJ software. The correlation index between cell resistance (days) and FoxO3A protein levels was calculated as:

$${\rm{correl}}\left( {X,Y} \right) = \frac{{{\sum} {\left( {x - x_m} \right)} \ast \left( {y - y_m} \right)}}{{\sqrt {{\sum} {\left( {x - x_m} \right)^2 \ast \sqrt {{\sum} {\left( {y - y_m} \right)^2} } } } }}$$

### Statistical analysis

Results are expressed as mean ± SEM and *n* ≥ 3. Student’s *t* test was used to define *p* values. A *p < 0.05, **p < 0.01, and ***p < 0.001 were considered statistically significant. Multiple comparisons are accounted for via the Hochberg’s method using PROC MULTTEST in SAS, as previously described^20^.

## Electronic supplementary material

Supplementary Figure Legends

Suppl Fig 1

Suppl Fig 2

Suppl Fig 3 - 4

Suppl Fig 5

Suppl Fig 6

Suppl Fig 7

Suppl Table 1 - 2
