# Supplementary material for: Uncoupling FoxO3A mitochondrial and nuclear functions in cancer cells undergoing metabolic stress and chemotherapy
Source: Cell Death Dis. 2018 Feb 14;9(2):231. doi: 10.1038/s41419-018-0336-0 (PMC5833443; doi:10.1038/s41419-018-0336-0)
Supplement: Supplementary file 1 — Supplementary Figure Legends [file 41419_2018_336_MOESM1_ESM.pdf]

Supplementary Figure S1. **(a)** Immunoblot analysis with the indicated antibodies of mitochondrial fractions isolated from HCT116 cells treated with iodoacetic acid (IA, 0,5 mM, 5h). Upon cellular fractionation, mitochondria were treated with proteinase K (PK). PDH was used as loading control. (**b)** Immunoblot analysis with the indicated antibodies of mitochondrial fractions isolated from LS174T human colorectal cancer cells, BT474 and EFM19 human breast cancer cells cultured in LG conditions (24h). Upon cellular fractionation, mitochondria were treated with PK alone to degrade outer mitochondrial membrane proteins or with PK and TritonX-100 to permeabilize mitochondria and degrade all mitochondrial proteins. BCL2: outer membrane control; PDH: mitochondrial matrix control. **(c)** Immunoblot analysis of different murine organs with the indicated antibodies. PDH was used as a loading control. The presented results are representative of at least three independent experiments.

Supplementary Figure S2. **(a)** Alignment of FoxO3A N-terminal sequence with cleavage consensus motifs for translocation into the mitochondrial matrix. R-2, XRXXX, and R-3, XRXXAX, are cleavage site motifs recognized by mitochondrial processing peptidase (MPP); the R-10 motif, XRXLXXGXXXX, is sequentially cleaved by MPP and mitochondrial intermediate peptidase (MIP). (**b)** Proteinase K (PK) protection assay scheme. Mitochondria were isolated as described in the Methods section. **(c)** Immunoblot analysis with the indicated antibodies of mitochondrial fractions isolated from HCT116 cells subjected to PK protection assay upon glucose restriction (LG, 0,75 mM glucose, 24h). **(d)** Immunoblot analysis with the indicated antibodies of mitoplasts obtained from HCT116 cells upon glucose restriction (LG, 24h). **(c, d)**. MCL1: outer membrane marker; CYTOCHROME C: inter-membrane space marker; COX4: inner membrane marker; VDAC1: inner membrane marker; HSP60: matrix marker. fl.: full-length FoxO3A; cl.: cleaved FoxO3A. The presented results are representative of at least three independent experiments.

Supplementary Figure S3. Alignment of the N-terminal region of human FoxO proteins performed using the CLUSTALW (1.83) tool available on the MSA (Multiple Sequence Alignment) server

(http://www.ebi.ac.uk/Tools/msa/). Human FoxO proteins were annotated with specific UniProtKB/Swiss-Prot accession numbers and entry names (http://www.uniprot.org).

Supplementary Figure S4. (a, b) Phylogenetic analysis showing homology of FoxO3 sequences from *Caenorhabditis elegans* to *Homo sapiens*. Sequences were aligned by using the T-Coffee tool available on the MSA (Multiple Sequence Alignment) server (http://www.ebi.ac.uk/Tools/msa/tcoffee/). **(a)** Multiple sequence alignment of the region encompassing residues S12 and S30. **(b)** Multiple sequence alignment of the region encompassing mitochondrial processing peptidase consensus motifs R-2, R-3 and R-10 (amino acid positions: 98-108).

Supplementary Figure S5. (a) *Left panel*: scheme of FoxO3A-WT-FLAG and mutated FoxO3A-FLAG plasmids. *Right panel*: immunoblot analysis with the indicated antibodies of total and mitochondrial proteins isolated from HCT116 cells transfected with the indicated plasmids for 48h and subjected to glucose restriction (LG, 0,75 mM glucose, 24h). TFAM was used as a mitochondrial fraction control. **(b)** Co-immunoprecipitation analysis with the indicated antibodies of mitochondrial fractions isolated from HCT116 cells transfected with FoxO3A-WT-FLAG for 48h and subjected to glucose restriction (LG, 24h). fl.: full-length FoxO3A; cl.: cleaved FoxO3A. The presented results are representative of at least three independent experiments.

Supplementary Figure S6. (a, b) *Left panel:* ChIP analysis of endogenous FoxO3A recruitment at FHRE #1-2 sites on mtDNA upon glucose restriction (LG, 0,75 mM glucose, 24h) in SW-480 **(a)** and HEK293 **(b)** cells. *Right panel*: mitochondrial gene regulation in SW-480 **(A)** and HEK293 **(B)** cells upon glucose restriction (LG, 24h). The dotted line corresponds to the expression levels detected in cells cultured in high glucose (HG). **(c, d)** HCT116-FoxO3A$^{-/-}$ cells were transfected with the indicated plasmids for 48h and treated with 2-deoxy-glucose (2-DG, 1 mM, 6h). **(c)** Mitochondrial

gene regulation assessed by RT-PCR. **(d)** Immunoblot analysis of total proteins with the indicated antibodies. HSP60 was used as loading control**. (a-c)** Black bars: *ATPase 6* and *8* genes; white bars: *COX1, COX2* and *COX3* genes; grey bars: *ND1, ND2, ND3, ND4, ND4L, ND5,* and *ND6* genes; light grey bar: *cytochrome b* gene. The presented results are representative of at least 3 independent experiments. Where applicable, data are presented as mean ± SEM and significance was calculated with Student's t test. *p value < 0.05 was considered statistically significant.

Supplementary Figure S7. (a, b, c) HCT116-FoxO3A$^{-/-}$ cells were transfected with the indicated plasmids for 48h. Upon treatment with 5-fluorouracil (5-FU, 2 μM, 24h) **(a)**, etoposide (VP-13, 40 μM, 24h) **(b)** or cisplatin (CDDP, 30 μM, 48h) **(c)**, relative cell viability and relative cell death were calculated. The presented results are representative of at least 3 independent experiments. Data are presented as mean ± SEM and significance was calculated with Student's t test. *p value < 0.05 and **p < 0.01 were considered statistically significant.
