# Supplementary figures and images for: Uncoupling FoxO3A mitochondrial and nuclear functions in cancer cells undergoing metabolic stress and chemotherapy
Source: Cell Death Dis. 2018 Feb 14;9(2):231. doi: 10.1038/s41419-018-0336-0 (PMC5833443; doi:10.1038/s41419-018-0336-0)

**a**

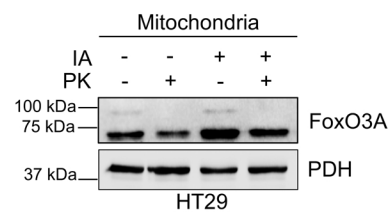

**b**

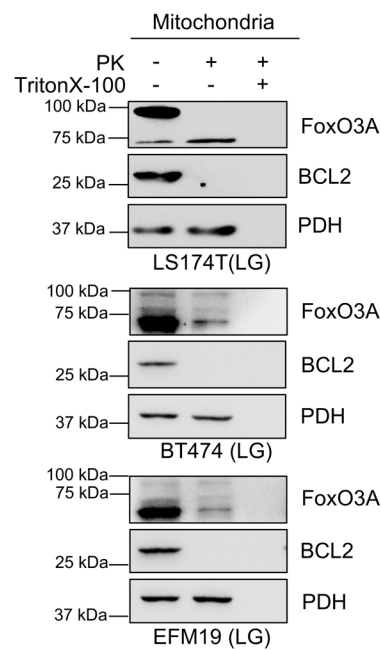

**c**

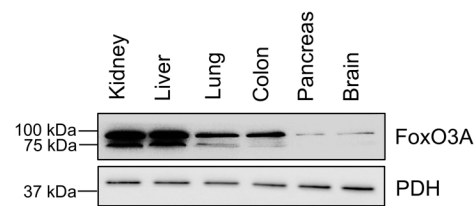

Supplement: Supplementary file 2 — Suppl Fig 1 [file 41419_2018_336_MOESM2_ESM.pdf]

**a**

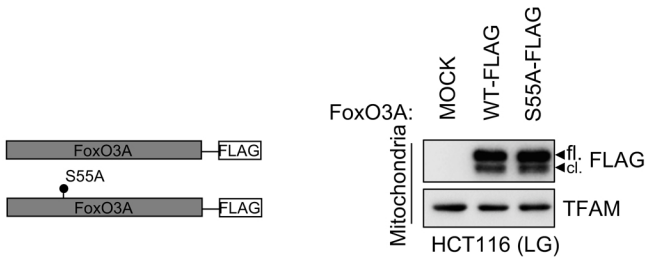

**b**

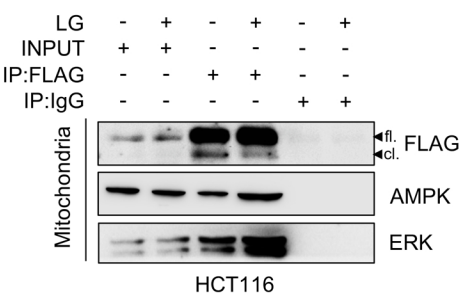

Supplement: Supplementary file 5 — Suppl Fig 5 [file 41419_2018_336_MOESM5_ESM.pdf]

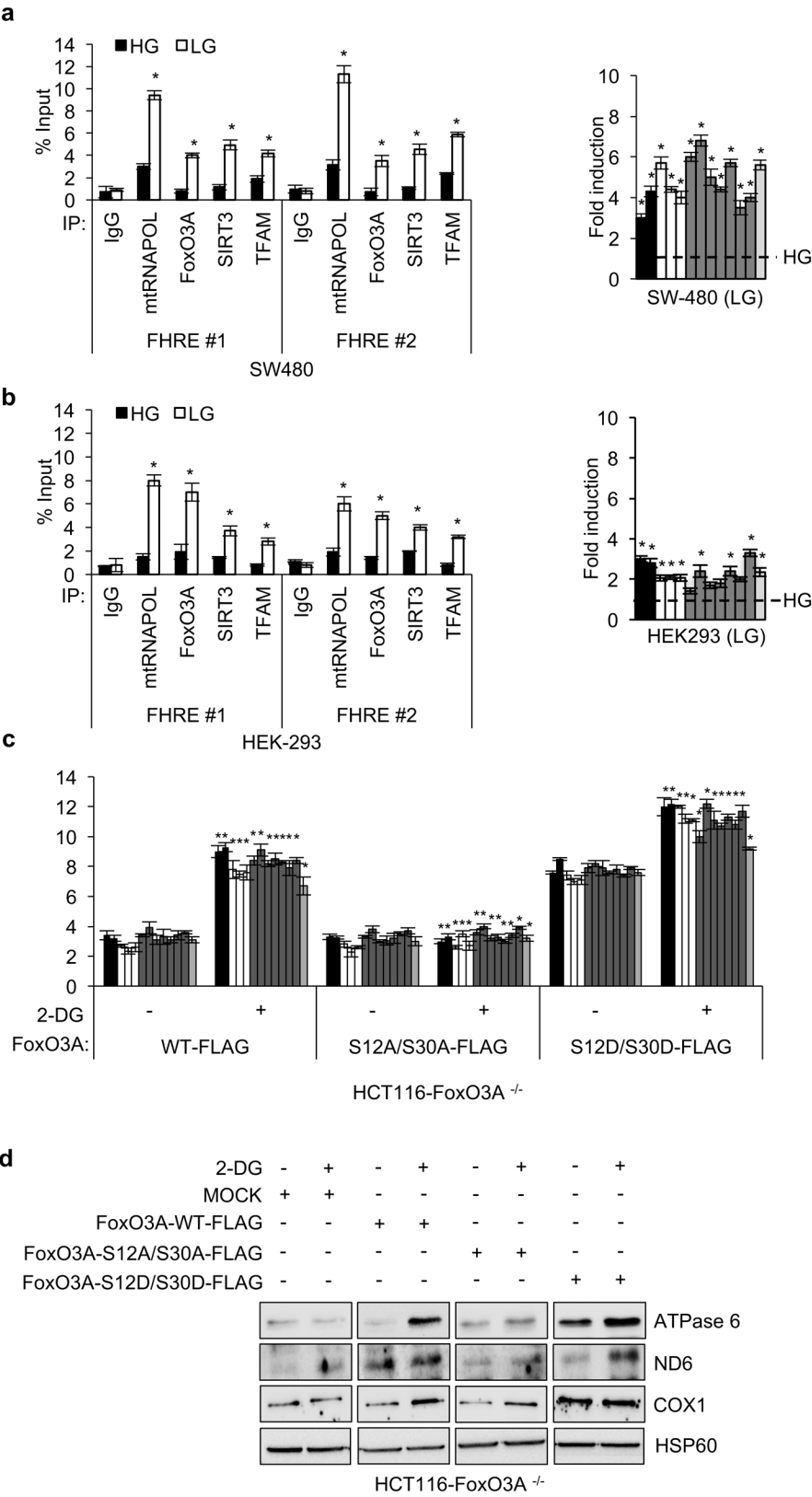

Supplement: Supplementary file 6 — Suppl Fig 6 [file 41419_2018_336_MOESM6_ESM.pdf]

**a**

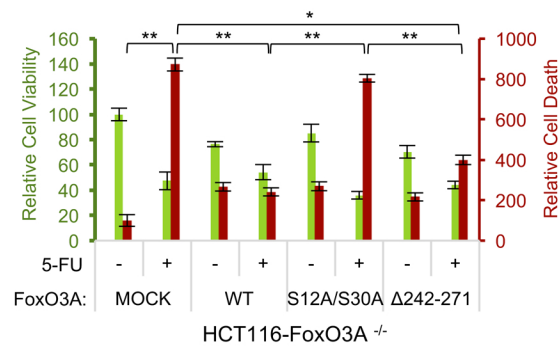

**b**

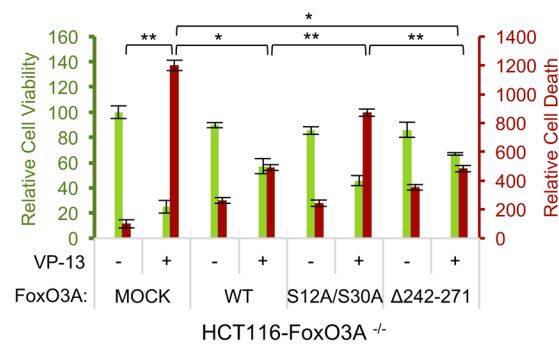

**c**

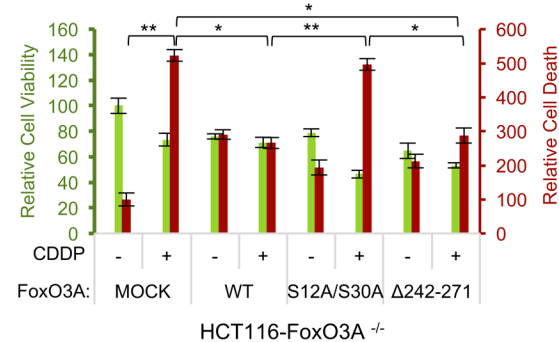

Supplement: Supplementary file 7 — Suppl Fig 7 [file 41419_2018_336_MOESM7_ESM.pdf]
