# Supplementary material for: Uncoupling FoxO3A mitochondrial and nuclear functions in cancer cells undergoing metabolic stress and chemotherapy
Source: Cell Death Dis. 2018 Feb 14;9(2):231. doi: 10.1038/s41419-018-0336-0 (PMC5833443; doi:10.1038/s41419-018-0336-0)

a

MPP/MIP Recognition motifs (R-2, R-3, R-10)

	80	90	100	110
**FoxO3A**	GGGGGSGTLGSGLLLEDSARVLAPGGQDPGS			
**R-2 motif**			XRXXX	
**R-3 motif**			XRXXAX	
**R-10 motif**			XRXLXXGXXXX	

b

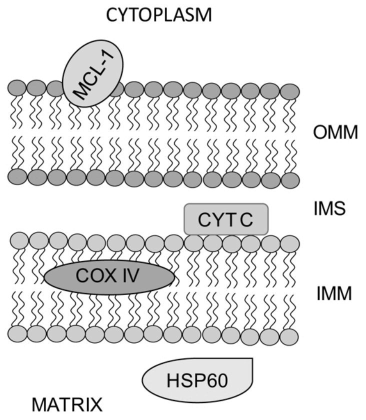

c

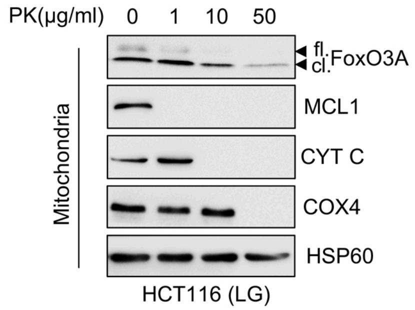

d

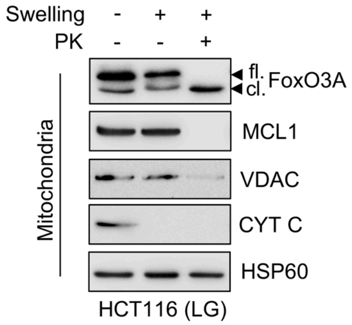

Supplement: Supplementary file 3 — Suppl Fig 2 [file 41419_2018_336_MOESM3_ESM.pdf]
