# Supplementary material for: Uncoupling FoxO3A mitochondrial and nuclear functions in cancer cells undergoing metabolic stress and chemotherapy
Source: Cell Death Dis. 2018 Feb 14;9(2):231. doi: 10.1038/s41419-018-0336-0 (PMC5833443; doi:10.1038/s41419-018-0336-0)
Supplement: Supplementary file 4 — Suppl Fig 3 - 4 [file 41419_2018_336_MOESM4_ESM.pdf]

Supplementary Figure_S3 Celestini et al. 2017

```
                       1         12                 30                 48
sP|O43524|FOXO3_HUMAN  MAEAPASPAPLSPLEVELDPEFEPQSRPRSCTWPLQRPELQASPAKPS--
sP|Q12778|FOXO1_HUMAN  MAE--------APQVVEIDPDFEPLPRPRSCTWPLPRPEFSQSNSATSSP
sP|P98177|FOXO4_HUMAN  MDPGNENSATEAAAIIDLDPDFEPQSRPRSCTWPLPRPEIANQPSEPPEV
sP|A8MYZ6|FOXO6_HUMAN  MA------AKLRAHQVDVDPDFAPQSRPRSCTWPLPQPDLAGDED-----
                       *        .  :::**:* * .********* :*::  .

                        R-2|----|
                        R-3|-----|
                    93 R-10|----------|                       134
sP|O43524|FOXO3_HUMAN  LLEDSARV-LAPGGQDPGSGPA---TAAGG-LSG----GTQALLQPQQPL
sP|Q12778|FOXO1_HUMAN  LLEESEDFPQAPGSVAAAVAAAAAAAATGG-LCGDFQGPEAGCLHPAPPQ
sP|P98177|FOXO4_HUMAN  ------------------LGEK---VHT--------EGRSEPILLPSRLP
sP|A8MYZ6|FOXO6_HUMAN  -----------------GALGAG---VAEGAEDCG----PERRATA---PA
                                        .     .
```

Supplementary Figure_S4 Celestini et al. 2017

a

```
                                          1          12                       30   35
Homo sapiens|FOXO3_HUMAN                  MAEAPASPAPLSP------------LEVELDPEFEPQSRPRSCT---WPL
Pan troglodytes|H2R710_PANTR              MAEAPASPAPLSP------------LEVELDPEFEPQSRPRSCT---WPL
Macaca mulatta|I2CWQ1_MACMU               MAEAPASPAPLSP------------LEVELDPEFEPQSRPRSCT---WPL
Ovis aries|I0B559_SHEEP                   MAEAPASPAPISP------------LEVELDPEFEPQSRPRSCT---WPL
Cricetulus griseus|G3GXK7_CRIGR           MAEAPASPVPLSP------------LEVELDPEFEPQSRPRSCT---WPL
Mus musculus|FOXO3_MOUSE                  MAEAPASPVPLSP------------LEVELDPEFEPQSRPRSCT---WPL
Pteropus Alecto|L5JMP7_PTEAL              MAEAPASPAPLSP------------LEVELDPEFEPQSRPRSCT---WPL
Ictalurus punctatus|W5UCF0_ICTPU          MAQASCEEKKPPT------------GDVDIDPDFEPQKRPRSCT---WPL
Drosophila melanogaster|FOXO_DROME        MMDGYAQEWPRLTHTDNGLAMDQLGGDLPLDVGFEPQTRARSNT---WPC
Caenorhabditis elegans|FOXO_CAEEL         MQLEQKSSLHCSK------------CRNFL-QKFSQDMQAWNCRELDSPL
                                          *    .                   :  *. : : . .     *
```

b

```
                                                                 R-2:XRXXS
                                                                 R-3:XRXXSX
                                          84                     R-10:XRXLXXGXXXX   116
Homo sapiens|FOXO3_HUMAN                  -----------------GSGTLGSGLLLEDSARVLAPGGQDPGSGPATAA
Pan troglodytes|H2R710_PANTR              -----------------GSGTLGSGLLLEDSARVLAPGGQDPGSGPATAA
Macaca mulatta|I2CWQ1_MACMU               -----------------GGGTLGSGLLLEDSARVLAPGGQDPGSGPATAA
Ovis aries|I0B559_SHEEP                   -----------------GGGPLGSGLLLEDSARLLAPGGQDPGSGPAPAA
Cricetulus griseus|G3GXK7_CRIGR           -----------------LSSTLGSGLLLEDSARLLAPGGQDLGSGPASAT
Mus musculus|FOXO3_MOUSE                  -----------------VSSTLGSGLLLEDSAMLLAPGGQDLGSGPASAA
Pteropus Alecto|L5JMP7_PTEAL              -----------------GCGTLGAGLLLEDSARLLAPGGQDPGSGPAPAA
Ictalurus punctatus|W5UCF0_ICTPU          T--S-YIRAKQSHNCDPPGPTLNTTCNDKDS-----DGSRSPSASQSLAA
Drosophila melanogaster|FOXO_DROME        ------------------------------SNQQLAPGDS----------
Caenorhabditis elegans|FOXO_CAEEL         TQYSQQFLREKC----SFSPYFHTSLETVDSGRTSLYGSNEQ-C--GQLG
                                                              *      *.
```
