# Supplementary material for: Uncoupling FoxO3A mitochondrial and nuclear functions in cancer cells undergoing metabolic stress and chemotherapy
Source: Cell Death Dis. 2018 Feb 14;9(2):231. doi: 10.1038/s41419-018-0336-0 (PMC5833443; doi:10.1038/s41419-018-0336-0)
Supplement: Supplementary file 8 — Suppl Table 1 - 2 [file 41419_2018_336_MOESM8_ESM.pdf]

Table S1. Prediction of phosphoprofile in FoxO3A human.

AA position	Sequence	[a]Netphos 2.0 score	[b]Disphos 1.3 score
[c]S12	PAPLSPLEV	0.98	0.757
[c]S26	FEPQSRPRS	0.6	0.754
[c]S30	SRPRSCTWP	0.97	0.775
[c]S43	ELQASPAKP	0.69	0.619
[c]S48	PAKPSGETA	0.86	0.645
[c]S55	TAADSMIPE	0.94	0.778
S75	GRAGSAMAI	0.483	0.810
S85	GGGGSGTLG	0.027	0.625
S90	GTLGSGLLL	0.006	0.359
S97	LLEDSARVL	0.039	0.410
S110	QDPGSGPAT	0.895	0.493
S120	AGGLSGGTQ	0.137	0.324
S144	AAGGSGQPR	0.013	0.416

[a] NetPhos 2.0 (http://www.cbs.dtu.dk/services/NetPhos/)
[b] Disphos prediction (Disorder-EnhAnced Phosphorylation Sites Predictor, http://www.dAbi.temple.edu/disphos/)
[c] All kinase orphan residues that show threshold score >0.6.

Table S2. Similarity aminoacids position between S30 motif in FoxO3A and AMPK consensus phosphorylation motif.

AMPK substrates	-5	-4	-3	-2	-1	0	+1	+2	+3	+4	+5
O43524 (FoxO3A)	Q	S	R	P	R	$\mathbf{S_{30}}$	C	T	W	P	L
Q9UHR4 (BI2L1)	P	S	L	Q	R	$\mathbf{S_{329}}$	V	S	V	A	T
Q96RT1 (LAP2)	K	N	I	V	R	$\mathbf{S_{913}}$	K	S	A	T	L
Q92974 (ARHG2)	L	A	K	S	V	$\mathbf{S_{151}}$	T	T	N	I	A
P20020 (AT2B1)	K	P	E	S	R	$\mathbf{S_{1176}}$	S	I	H	N	F
O14683 (P5I11)	L	M	K	K	H	$\mathbf{S_{14}}$	Q	T	D	L	V
P49815(TSC2)	L	S	K	S	S	$\mathbf{S_{1387}}$	S	P	E	L	Q

Supplementary Table S1-S2. Phospho-profile prediction in FoxO3A human sequence using NetPhos 2.0 (http://www.cbs.dtu.dk/services/NetPhos/) and Disphos 1.3 (Disorder-EnhAnced Phosphorylation Sites Predictor, http://www.dAbi.temple.edu/disphos/) servers. A threshold score > 0.6 with both tools was considered significant.
